# Molecular adaptation and resilience of the insect’s nuclear receptor USP

**DOI:** 10.1186/1471-2148-12-199

**Published:** 2012-10-05

**Authors:** Arnaud Chaumot, Jean-Luc Da Lage, Oscar Maestro, David Martin, Thomas Iwema, Frederic Brunet, Xavier Belles, Vincent Laudet, François Bonneton

**Affiliations:** 1 Institut de Génomique Fonctionnelle de Lyon (IGFL), Université de Lyon, Université Lyon 1; CNRS; INRA; Ecole Normale Supérieure de Lyon, 32-34 avenue Tony Garnier, Lyon, 69007, France; 2 Irstea, UR MALY, Lyon, F-69336, France; 3 UPR9034, Laboratoire Evolution, génomes et spéciation (LEGS), CNRS, Gif sur Yvette, 91198, France; 4 Institute of Evolutionary Biology (CSIC-UPF), Passeig Marítim de la Barceloneta 37, Barcelona, 08003, Spain; 5 Groupe de recherche "immunopathologie et maladies infectieuses (GRI), Universite de la Réunion, Centre CYROI, Cyclotron Réunion Ocean Indien, Sainte Clotilde Ile de la Réunion, 97491, France

**Keywords:** Nuclear receptors, Ecdysone receptor, ECR, USP, Mecopterida, Selection

## Abstract

**Background:**

The maintenance of biological systems requires plasticity and robustness. The function of the ecdysone receptor, a heterodimer composed of the nuclear receptors ECR (NR1H1) and USP (NR2B4), was maintained in insects despite a dramatic divergence that occurred during the emergence of Mecopterida. This receptor is therefore a good model to study the evolution of plasticity. We tested the hypothesis that selection has shaped the Ligand-Binding Domain (LBD) of USP during evolution of Mecopterida.

**Results:**

We isolated *usp* and *cox1* in several species of Drosophilidae, Tenebrionidae and Blattaria and estimated non-synonymous/synonymous rate ratios using maximum-likelihood methods and codon-based substitution models. Although the *usp* sequences were mainly under negative selection, we detected relaxation at residues located on the surface of the LBD within Mecopterida families. Using branch-site models, we also detected changes in selective constraints along three successive branches of the Mecopterida evolution. Residues located at the bottom of the ligand-binding pocket (LBP) underwent strong positive selection during the emergence of Mecopterida. This change is correlated with the acquisition of a large LBP filled by phospholipids that probably allowed the stabilisation of the new Mecopterida structure. Later, when the two subgroups of Mecopterida (Amphiesmenoptera: Lepidoptera, Trichoptera; Antliophora: Diptera, Mecoptera, Siphonaptera) diverged, the same positions became under purifying selection. Similarly, several positions of the heterodimerisation interface experienced positive selection during the emergence of Mecopterida, rapidly followed by a phase of constrained evolution. An enlargement of the heterodimerisation surface is specific for Mecopterida and was associated with a reinforcement of the obligatory partnership between ECR and USP, at the expense of homodimerisation.

**Conclusions:**

In order to explain the episodic mode of evolution of USP, we propose a model in which the molecular adaptation of this protein is seen as a process of resilience for the maintenance of the ecdysone receptor functionality.

## Background

During the course of evolution, the maintenance of biological systems requires the conservation of vital functions. At the same time, living beings are facing constant changes in their genome and environment. These variations, which are continuously challenging the fitness of the organism, are key modulators of adaptation. It is therefore essential to maintain a good balance between plasticity and robustness [1]. How does this equilibrium evolve at the genetic level? In order to tackle this important issue, it is necessary to use a model system with a vital function that has been conserved during a given period of time, despite the divergence of the genetic modules that determine this function. The ecdysone receptor fulfils these requirements. This hormone receptor is a heterodimer composed of two nuclear receptors: ECR (NR1H1) and its obligatory partner USP (NR2B4), the ortholog of mammalian RXR (NR2B1,2,3). The activation of ECR by ecdysteroid hormones triggers a gene cascade that controls development (embryogenesis, molts, metamorphosis) and reproduction in insects [2,3]. Both partners of this hormone receptor have undergone a dramatic divergence during the emergence of the Mecopterida, a superorder of holometabolous insects, which includes Diptera (flies, mosquitoes), Lepidoptera (moths, butterflies), Mecoptera (scorpionflies), Siphonaptera (fleas), and Trichoptera (caddisflies) [4-6]. This acceleration of evolutionary rate is correlated with structural and functional changes of the LBP of USP and of the heterodimer interface between ECR and USP [7,8]. These results suggested to us that the maintenance of the ecdysone pathway was achieved, at least in part, through molecular adaptation of the USP LBD. We thus tested the hypothesis that selection has shaped this domain during the emergence of Mecopterida.

Changes in evolutionary rates are commonly used to detect innovations in the function of proteins. It is generally assumed that a slowdown of substitution rate is caused by the action of negative purifying selection that maintains an important function. By contrast, an acceleration of evolutionary rate can be interpreted in two opposite ways. It could be due to a phase of positive selection on a new function, or to neutral evolution of a functionless sequence. This simplistic approach raises several problems. Indeed, the rate of evolution at a given position can change according to its interactions with other sites. Many interactions are compatible with different conformations of a protein with the same function. Therefore, within-site rate variation in time (heterotachy) is not necessarily associated with a change in function, but may simply reflect the epistatic nature of genetic units [9]. In other words, the same site of a protein can follow different evolutionary paths in a phylogenetic tree, and whether these variations are adaptive or neutral cannot be inferred solely from the estimate of evolutionary rates. It is essential to take into account the interdependence of residues, within and between proteins. Consequently, new methods try to incorporate protein structural constraints into evolutionary models [10]. Another important problem is to distinguish between positive selection and relaxation of negative selection [11]. Furthermore, the evolution of protein function involves alternating periods of conservative evolution and of rapid change [12]. As a consequence, evidence for positive selection at the protein level are rare at a short time scale but much more frequent when considering deeper evolutionary time [13]. Finally, non-adaptive processes such as variations of the mutation rate or GC-biased gene conversion, can lead to an increase of evolutionary speed that is independent of natural selection [14,15]. We are trying to tackle some of these issues that are central for a correct understanding of the genetic mechanisms of protein evolution using the ecdysone receptor. Thanks to the availability of crystal structures and functional data, the ECR-USP case provides clear biological hypotheses regarding the codons upon which positive selection is expected. Using appropriate models, we could test our hypotheses at different time scales by analysing evolutionary constrains either within extant insect families, or during three successive branches of Mecopterida radiation. Our results reveal the dynamics of evolutionary forces acting on the LBD of USP.

## Results

### Sequence variation of *usp* and *cox1* in three insect species-goups

**Figure 1:**
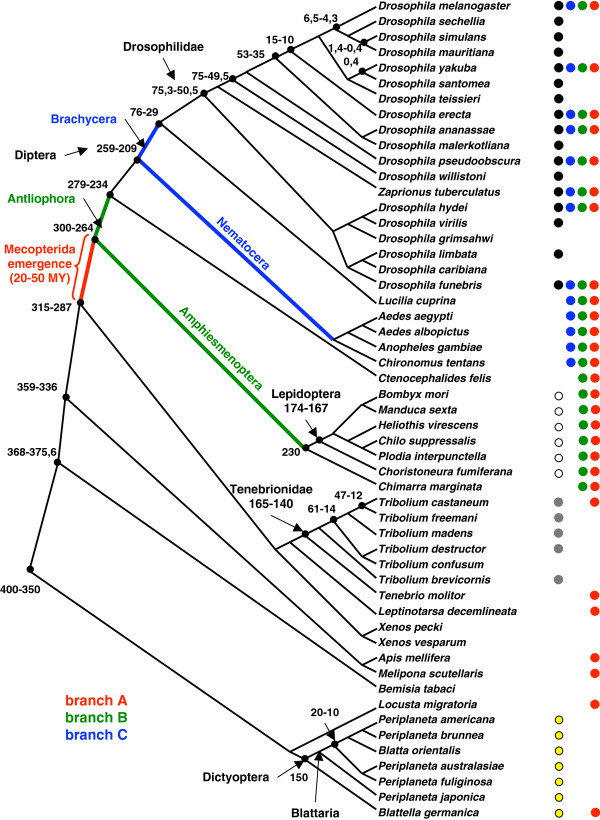
**Phylogeny of insects species used in this study.** This consensus tree and the divergence times (million years) are based on the following references: Drosophilidae [16,66], Diptera [65], Lepidoptera [67,68], Tenebrionidae [17], Blattaria [18,19,69] and insects [70-72]. Dots indicate the species used for detection of selection in *usp* along different branches. Black dots: Drosophilidae; blue dots: Diptera (branch C); green dots: divergence between Antliophora and Amphiesmenoptera (branch B); red dots: emergence of Mecopterida (branch A); white dots: Lepidoptera; grey dots: Tenebrionidae; yellow dots: Blattaria.

In order to study the evolutionary dynamics of USP at different time scales, we isolated sequences from several species of Drosophilidae, Tenebrionidae and Blattaria (Figure 1; Additional file 1: Table S1). We have chosen the first two families as representative of the holometabolous insects, with Drosophilidae showing the Mecopterida “overacceleration” of USP [4,6]. Tenebrionidae belong to Coleoptera, which is the sister group of Mecopterida, while Blattaria are hemimetabolous insects. Both Tenebrionidae and Blattaria have a non-divergent USP [4]. The 19 Drosophilidae species used in this study span an evolutionary time of 0.4 to 75 million years [16]. The most ancient divergence time for the five true *Tribolium* species used in our analysis was estimated between 14 and 61 million years ago [17]. Blattaria species may have diverged between 10 and 20 million years ago [18,19].

In order to compare the variable rates of USP evolution between these different insect groups, we used the gene *cox1*, which encodes the mitochondrial cytochrome oxidase subunit 1. This gene is known to evolve under a relatively uniform rate in most taxa of insects [20]. Overall, our data set consists of protein sequences for both USP and COX1 in 32 species of Drosophilidae, Tenebrionidae and Blattaria (Additional file 1: Table S1). Among these 64 sequences, 43 are new and were isolated for this study. The alignment of these proteins provides 218 positions (including gaps) for USP LBD and 410 positions (no gap) for COX1. As expected, the COX1 protein sequences are very well conserved between these three insect groups (>85% identity), whereas the USP LBD of Drosophilidae is highly divergent from the other species (≈40% identity).

The phylogenies that we obtained using alignments of concatenated sequences (1042 positions) of USP and COX1 proteins are similar to those already published (data not shown). For example, we observed that *Tribolium brevicornis* is not closely related to the other *Tribolium* species, as shown by Angelini *et al.*[17], who suggest that it should be included into the genus *Aphanotus*. We also observed the paraphyly of *Periplaneta spp.*, with *Blatta orientalis* located within this clade, as shown by other authors [18,19].

### The negative selection on the *usp* gene is weaker in Mecopterida families

We estimated non-synonymous/synonymous rate ratios (*d*_N_/*d*_S_ or *ω*) by whole tree analyses using maximum-likelihood methods and codon-based substitution models [21,22] (Additional file 2: Table S3). We analysed three groups of species that are characterized by a derived *usp* (Drosophilidae, Diptera, Lepidoptera) and two groups with a less modified *usp* (Tenebrionidae and Blattaria) (Figure 1).

**Figure 2:**
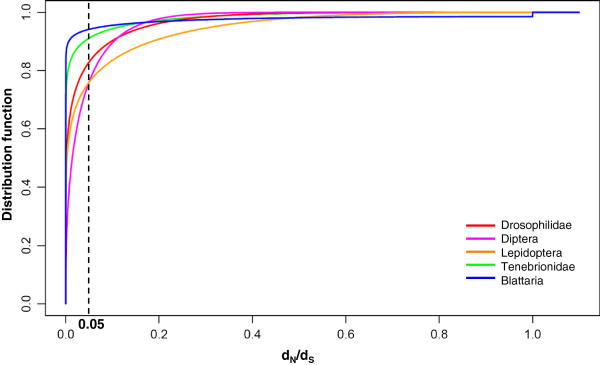
**Distribution function of *d***_**N**_**/*d***_**S**_**in *usp *for five extant groups of insects.** The curves describe the estimated proportion of codons that have *d*_N_/*d*_S_ ratios at a value less than or equal to a particular value on the abscissa. β distributions were compared by Kolmogorov-Smirnov tests. The β-distribution parameters were estimated for the M7 model, or M8 if statistically superior to M7 (*see* Table 2). Drosophilidae: red; Diptera: purple; Lepidoptera: orange; Tenebrionidae: green; Blattaria: blue.

If we consider each group separately, the best model indicates that the main force acting on *usp* sequences is negative selection: *d*_N_/*d*_S_ is less than 0.05 for at least 75% of the sites (Figure 2). We could detect rare neutral codons in Blattaria (3/279), but not in the other groups (Table 1). Likelihood ratio tests contrasting positive selection models with nearly neutral models (M2a *versus* M1a or M8 *versus* M7) never conclude that positive selection (*d*_N_/*d*_S_ > 1) is acting on *usp* within these groups (Table 1). This notably explains the difficulty to adjust parameters of models M2a and M8, as shown by standard errors associated to the estimation of *p*_*1*_*, ω*_2_ and *ω*_s_ (Additional file 3: Table S4). The highly constrained pattern also gives rise to an imprecise estimation (large relative standard errors) of the two shape parameters of the β distribution for the two non-Mecopterida groups (Additional file 3: Table S4). This is coherent with the very weak improvement of the likelihood between M1a and M7/M8 models for these two groups. Kolmogorov-Smirnov tests indicate significant differences in the distribution of site-specific *d*_N_/*d*_S_ between insect groups (p-value < 10^-7^). As an illustration, the percentage of codons with a *d*_N_/*d*_S_ above 0.05 is 20% in Mecopterida and only 5% in non-Mecopterida groups (Figure 2). Since saturation was detected among Diptera *usp*, we performed a re-analysis of alignments simulated according to the estimated characteristics of the Diptera dataset (13 species, 346 codons, same topology and branch lengths for the phylogenetic tree, same codon usage, transition/transversion rate ratio of 1.89, repartition of sites between ten categories of *d*_N_/*d*_S_ according to the estimated β distribution). The results showed a good accuracy in the estimate of the β distribution function (Additional file 4: Figure S1), indicating that the detection of this pattern is robust to the saturation in Diptera sequences. The confidence interval for the proportion of codons with *d*_N_/*d*_S_>0.05 was less than ± 1%. In the same way, we confirmed that the weak precision in the estimation of the β distribution parameters for the Tenebrionidae and Blattaria does not alter their contrasted pattern with Mecopterida groups (data not shown). The Mecopterida specific regions are among the less constrained of *usp* because, when they are removed from the analysis, the Mecopterida curves shift slightly towards Tenebrionidae and Blattaria curves (data not shown). To compare the evolutionary dynamics of *usp* between groups, we used *cox1* as a reference (410 codons) (Additional file 5: Table S5). In Drosophilidae, the mean rate of non-synonymous substitutions of *usp* is 3.4 times higher than for *cox1*. By contrast, *usp* accumulates less non-synonymous substitutions than *cox1* in the non-Mecopterida families (2.5 times less in Tenebrionidae and 1.3 times less in Blattaria). Overall, by employing *cox1* as a relative molecular clock, the Drosophilidae *usp* gene evolved 8.6 times faster than the Tenebrionidae *usp* and 4.4 times faster than the Blattaria homolog.

**Table 1:** **Likelihood analysis of the *usp *sequence datasets of five extant insect groups**

**Model**		**Drosophilidae**	**Diptera**	**Lepidoptera**	**Tenebrionidae**	**Blattaria**
M1a	Ln*L (np)*	-5649.5 *(31)*	-6946.6 *(25)*	-4376.2 *(12)*	-2113.5 *(10)*	-1945.6 *(13)*
	*ω*_*0*_*ω*_*1*_	0.02 1.00	0.03 1.00	0.03 1.00	0.01 1.00	0.00 1.00
	*p*_*0*_*p*_*1*_	0.98 0.02	0.98 0.02	0.95 0.05	0.99 0.01	0.99 0.01
M2a	Ln*L (np)*	-5649.5 (*33*)	-6946.6 *(27)*	-4376.2 *(14)*	-2113.5 (*12*)	-1945.6 (*15*)
	*ω*_*0*_*ω*_*1*_*ω*_*2*_	0.02 1.00 1.00	0.03 1.00 1.00	0.03 1.00 1.00	0.01 1.00 1.00	0.00 1.00 1.24
	*p*_*0*_*p*_*1*_*p*_*2*_	0.98 0.01 0.01	0.98 0.01 0.01	0.95 0.03 0.02	0.99 0.01 0.00	0.99 0.01 0.00
M7	Ln*L (np)*	-5610.8 *(31)*	-6859.5 *(25)*	-4339.9 *(12)*	-2112.9 *(10)*	-1950.0 *(13)*
	*p q*	0.16 5.05	0.40 10.7	0.15 2.58	0.06 3.13	0.01 0.31
M8	Ln*L (np)*	-5610.8 *(33)*	-6857.1 *(27)*	-4338.8 *(14)*	-2112.9 *(12)*	-1946 *(15)*
	*p q*	0.16 5.05	0.43 12.9	0.16 2.85	0.06 3.13	0.02 2.09
	*ω*_*s*_	1.00	1.00	4.46	1.00	1.00
	*p*_*s*_	0.00	0.01	0.00	0.00	0.01
LRT (p-value)		*test of positive selection*				
M2a *vs* M1a		1	1	1	1	1
M8 *vs* M7		1	0.08	0.32	1	0.02

Ln*L*: log likelihood. np: number of parameters. *ω*_*0*_*, ω*_*1*_*, ω*_*2*_: non-synonymous/synonymous rate ratio that characterize a negative, a neutral, and a positive selection regime, respectively (with corresponding proportions of sites *p*_*0*_*, p*_*1*_*, p*_*2*_). *p* and *q:* shape parameters of a β function describing the distribution of non-synonymous/synonymous rate ratios. *ω*_*s*_: ratio affecting a *p*_*s*_ proportion of sites under positive selection in model M8. LRT: Likelihood ratio test.

These results suggest that negative selection on *usp* is relaxed in Mecopterida families, when compared to non-Mecopterida families.

### Relaxation of negative selection on the external surface of USP protein

**Figure 3:**
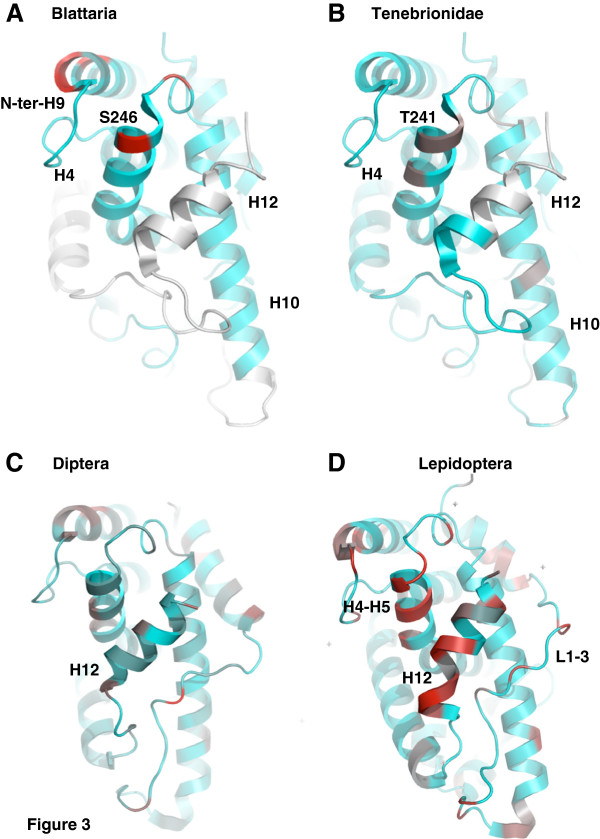
**Mapping of evolutionary rates on USP structures in four extant groups of insects.** Site-specific *d*_N_/*d*_S_ were projected onto the crystal structure of the USP LBD domain of *Tribolium* (2NXX) for Blattaria (**A**) and Tenebrionidae (**B**), of *Drosophila* (1HG4) for Diptera (**C**) and of *Heliothis* (1R1K) for Lepidoptera (**D**). The values are distributed along a colour scale from blue (low *d*_N_/*d*_S_) to red (high *d*_N_/*d*_S_). Sequences not available for the estimation of evolutionary rates are in white.

In order to identify precisely the sites that present contrasted patterns in selective constraints between these groups, we projected the site-specific *d*_N_/*d*_S_ onto the available 3D structures of USP protein (Figure 3). Three functional domains of USP are highly constrained in every group of insects: the DBD (Additional file 2: Table S3), the LBP and the dimerisation interface. By contrast, other parts of the protein have experienced more relaxed evolutionary pressure, especially among the Mecopterida families.

The LBD of non-Mecopterida USPs are homogeneously constrained. Only 9 sites (5%), located on the external surface of the LBD, have an estimated *d*_N_/*d*_S_ ≥ 0.1 in Tenebrionidae and Blattaria. The uncertainty in parameter estimates (Additional file 3: Table S4) for these two groups may hinder the computation of site-specific rates, but these sites are not homologous in these two insect groups, except one position in helix H4 (Ser246 in Blattaria and Thr241 in Tenebrionidae) (Figure 3A, B).

The LBD of Mecopterida USPs shows very different patterns, with 63 sites (25%) having a *d*_N_/*d*_S_ ≥ 0.1. Overall, the relaxation of evolution is more pronounced in Lepidoptera than in Diptera. Only one-third of these sites are similar between these two insect orders. Most of the relaxed residues are scattered on the surface of the LBD (Additional file 6: Figure S2), a few of them being located in regions with a known or putative function. For instance, the hinge D domain and the following helix H1 of the LBD exhibit a high proportion of relaxed positions in Diptera and Lepidoptera (17/30 and 8/28, respectively), when compared to Tenebrionidae and Blattaria (1/31 and 0/31, respectively) (Additional file 2: Table S3). This region is known to have an allosteric effect on the corepressor-binding surface of RXR and other nuclear receptors [23,24]. A cluster of seven relaxed positions is shared by both groups of insects in helix H4-H5, which is included into the putative coactivation surface (Figure 3C, D). In helix H12, the Lepidoptera sequences are totally divergent from all other USP-RXR proteins. Contrary to ECR, the helix H12 of USP has no transactivation activity because it adopts a structure similar to the antagonist conformation observed for RXR [7,25-28]. In *Drosophila* and in *Heliothis*, helix H12 is locked into this position by the Mecopterida specific domain present in the loop L1-3, between helices H1 and H3. Interestingly, several residues of this specific insertion are evolutionary relaxed in Diptera, and even more in Lepidoptera (Figure 3C, D). A similar pattern of relaxation seems to occur in the loop between helix H5 and the first beta turn (Additional file 2: Table S3), where there is a second Mecopterida specific insertion of 20–30 amino acids. This divergent region, only partly stabilized in the crystal structures of *Heliothis* and *Drosophila*, is possibly implicated in contacts with ECR [25,29]. By contrast, the residues that constitute the heterodimerisation interface are constrained in Diptera and Lepidoptera.

These results show that the main feature of the recent evolution of USP is a relaxation of purifying selection in Mecopterida families. The LBP and the dimerisation interface, which underwent important Mecopterida specific changes in structure and function [7,8], are highly constrained at this time scale and positive selection was not detected. By contrast, an evolutionary relaxation might have affected external surfaces involved in protein-protein interactions between USP and its coregulators.

### Positive selection on the *usp* gene during the emergence of Mecopterida

Since USP LBD underwent important modifications in Mecopterida, we wondered whether some of these changes involved functional shifts during the emergence of this group. Assuming that evolutionary pressure is changing over time, we used branch-site models [30] to detect potential site-specific changes in selective constraints (positive selection or relaxation of negative selection) along three successive branches of Mecopterida radiation. Branch A is the stem lineage of Mecopterida, branch B is the subdivision between Amphiesmenoptera and Antliophora and branch C is the subdivision of Diptera into the suborders Brachycera and Nematocera (Figure 1).

In order to assess whether relaxation or positive selection occurred along these branches, we applied tests 1 and 2 from Zhang *et al.*[30]. While test 1 is significant for all the three branches, test 2, a direct conservative test of positive selection, is significant only for the two most ancient branches A and B (Table 2). It appears therefore that positive selection has occurred on specific sites at the origin of the Mecopterida clade (21% of *usp* sites) and during the divergence between Amphiesmenoptera and Antliophora (11% of *usp* sites). Subsequently, some sites (8%) evolved neutrally before becoming under purifying selection within Dipteran suborders (Nematocera and Brachycera). Since the specificity of these tests could be affected by long evolutionary distances, false positive rates were computed, as a control, from the re-analysis of alignments simulated with Evolver in PAML [12]. These rates were less than 1% for test 1 for the three datasets. For test 2, we obtained false positive rates of 5% for branch A and 2% for branch B. Therefore, our detection of positive selection is not significantly biased by saturation of synonymous sites during the large evolutionary times considered in this analysis. The initial episode of positive selection was dramatic, if we consider the percentage of sites involved and the high values of *d*_N_/*d*_S_ for the site class under positive selection (ω_2_ of branch B in Table 2). Note that the value of 999 computed for ω_2_ along the stem lineage of Mecopterida (branch A) indicates the absence of predicted synonymous substitutions for this short branch (*d*_S_=0), so the *d*_N_/*d*_S_ ratio (ω_2_) is undefined.

**Table 2:** **Likelihood analysis of the *usp *sequence datasets along three successive branches of the Mecopterida radiation**

	**Model**				**Likelihood Ratio Test**	
	**No Shift**	**Branch relaxation**	**Branch positive selectionBranch positive selection**		**Relaxation**	**positive selection**
	**M1a *(nearly neutral)***	**modified branch-site model A ω**_**2**_** = 1 fixed**	**modified branch-site model A**		**test 1**	**test 2**
	Ln*L (np)*	**Ln*L (np)***	Ln*L (np)*	*ω*_*2*_	p-value	p-value
**Branch A**	-11732.4 *(55)*	-11713.2 *(56)*	-11711.1 *(57)*	999	6.6 10^-11^	**4.2 10**^**-2**^
**Branch B**	-9769.8 *(41)*	-9754.5 *(42)*	-9750.9 *(43)*	97.3	8.0 10^-9^	**6.8 10**^**-3**^
**Branch C**	-6946.6 *(25)*	-6938.1 *(26)*	-6937.5 *(27)*	4.75	1.1 10^-4^	0.56

Ln*L*: log likelihood. *np*: number of parameters.

The sites that experienced a functional shift along the three branches were identified by computing the probability for each site to belong to the site-class that experienced positive selection (along branch A or branch B) or neutral evolution (along branch C), before becoming under negative selection in extant groups (Additional file 7: Table S6). The projection of these posterior probabilities onto the 3D structure of the LBD of *Drosophila* USP reveals that most of these sites under positive selection, or relaxation, are on the external surface of the LBD, notably on branch A. There is no known function for the majority of these sites. A minority of the positively selected amino acids are located on two functional domains of the LBD that are the LBP and the heterodimerisation interface: 36% on branch A, 15% on branch B and 21% on branch C. Among the residues with a known function, there is a high fraction which is positively selected or relaxed on branch A (18/36), and less for branch B (7/20) and branch C (6/14).

**Figure 4:**
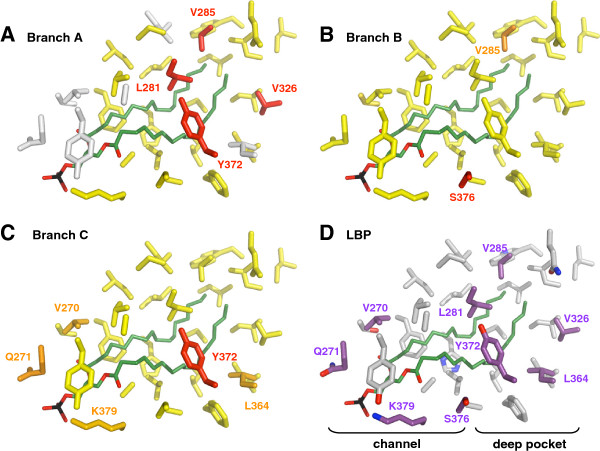
**Selection in the LBP during the radiation of Mecopterida.** Posterior probabilities for each site to belong to the site-class under positive selection (along branch **A** or branch **B**) or relaxed evolution (along branch **C**) were projected onto the crystal structure of the USP LBD domain of *Drosophila* (1HG4). Probabilities are distributed along a colour scale from yellow (p=0) to red (p=1). Sequences not available for the estimation of evolutionary rates are in white. The phospholipid ligand (LPP) is in green, with its tail on the right in the deep pocket. (**A**) Branch **A**, stem lineage of Mecopterida. (**B**) Branch **B**, subdivision between Amphiesmenoptera (Lepidoptera, Trichoptera) and Antliophora (Diptera, Mecoptera, Siphonaptera). (**C**) Branch **C**, subdivision of Diptera into the suborders Brachycera and Nematocera. (**D**) All the 32 amino acids of the LBP in the USP structures of *Drosophila* and *Heliothis*. Residues under positive selection or relaxed evolution in at least one of the three branches are shown in purple.

The large LBP of Mecopterida USP is composed of two parts: a channel extending inside from the opening and a deeper pocket, which is similar to the LBP of RXR. The tail of the phospholipid is buried inside this pocket, while its phosphate head group is at the entrance. The amino acids of the channel, from where the ligand penetrates inside the LBP, are under negative selection in the three branches. By contrast, four residues that make contacts with the ligand at the bottom of the internal surface underwent strong positive selection during the emergence of Mecopterida (Figure 4A). This change of evolutionary pressure is correlated with the specific acquisition of a large LBP filled by phospholipids in Mecopterida. In other insects, residues of the loops L6 and L11 fill the deep unliganded pocket. Later, when Amphiesmenoptera and Antliophora diverged (branch B), the same positions became under purifying selection (Figure 4B). Subsequently, one of them (Tyr372), followed a new path of relaxed evolution during the subdivision of Dipteran suborders (branch C) (Figure 4C). All Brachycera share a tyrosine at this position, while Nematocera and other Mecopterida have a leucine. In the homologous position of RXR there is a valine, which does not interact with the ligand.

**Figure 5:**
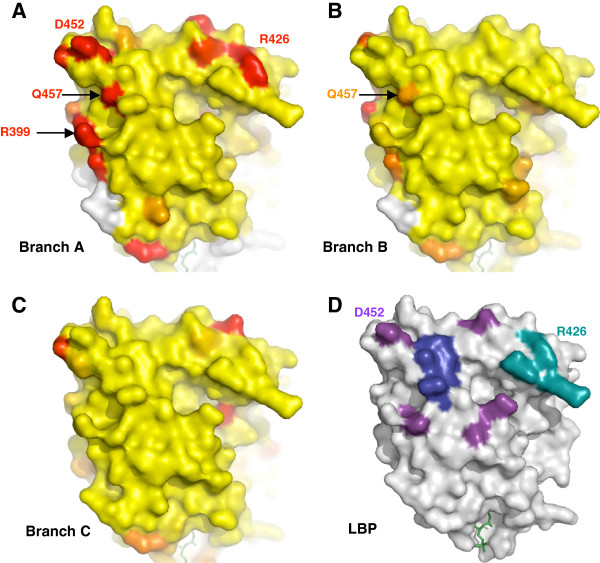
**Selection in the heterodimerisation interface during the radiation of Mecopterida.** Posterior probabilities for each site to belong to the site-class under positive selection (along branch **A** or branch **B**) or relaxed evolution (along branch **C**) were projected onto the crystal structure of the USP LBD domain of *Drosophila* (1HG4). Probabilities are distributed along a colour scale from yellow (p=0) to red (p=1). Sequences not available for the estimation of evolutionary rates are in white. The lipid ligand (LPP) is in green. (**A**) Branch **A**, stem lineage of Mecopterida. (**B**) Branch **B**, subdivision between Amphiesmenoptera (Lepidoptera, Trichoptera) and Antliophora (Diptera, Mecoptera, Siphonaptera). (**C**) Branch **C**, subdivision of Diptera into the suborders Brachycera and Nematocera. (**D**) The hydrophobic core (helix H10) is shown in blue, the insect canonical interaction surface is in purple and the Mecopterida specific surface is in cyan (for details, see Additional file 1: Table S1 of: [8].

A similar evolutionary dynamic is observed for the heterodimerisation interface, where several positions experienced positive selection during the emergence of Mecopterida, followed by a phase of constrained evolution (Figure 5). Importantly, among these positions, arginine Arg426 in helix H9 (Figure 5A) is known to be involved in the Mecopterida specific enlargement of the heterodimerisation surface [8]. On the same branch, positive selection is detected on three other residues that, albeit not interacting with ECR, are located within the canonical interaction surface. The arginine Arg399 in helix H7 was predicted to form an electrostatic interaction with ECR in the ancestral Mecopterida model [8]. This is interesting because this bond is not seen in the current USP structure of *Drosophila* or *Heliothis*[25,26]. Another noticeable residue is the glutamine Gln457 in helix H10, which is variable in Mecopterida. Indeed, its homologous position in non-Mecopterida USP and in vertebrate RXR is a strictly conserved lysine that interacts with the partner of heterodimerisation. This bond was observed in human and mouse RXR heterodimers as well as in the ECR-USP structure of the Hemiptera *Bemisia tabaci* white flies, but not in *Tribolium*[7,28].

By contrast to the LBP and to the dimerisation interface, there is no clear evidence of positive selection in the putative coactivation surface, simply defined here by homology with RXR (Additional file 8: Figure S3). Although we could not estimate the evolutionary rate for the whole surface in the three branches, it seems that this domain is rather homogeneously constrained. Only two residues in helix H3 have experienced positive selection during the emergence of Mecopterida (branch A), one of which being also selected on branch B (subdivision between Amphiesmenoptera and Antliophora).

In conclusion, the structural analysis of the sites under selection shows that both the LBP and the dimerisation interface of USP LBD have experienced modifications of selective constraints during the emergence of Mecopterida.

### Did biased gene conversion contribute to the early evolution of *usp* in Mecopterida*?*

Acceleration of substitution rates can result from other processes than selection, such as GC-biased gene conversion (gBGC), which enrich the G+C content [15,31]. We thus measured the G+C content of *usp* gene and found that it is higher in Drosophilidae (59.6%), when compared to Tenebrionidae and Blattaria (48.7%). The highest average G+C content is at third codon positions, where the Mecopterida G+C bias is stronger (Table 3). As a result, the preference for codons ending with a G or a C in *usp* is higher in Mecopterida than in non-Mecopterida for most of the amino acids (Additional file 9: Table S2). Therefore, the nucleotide composition of *usp* gene in Mecopterida is biased. By contrast, the average *cox1* G+C content are similar in the three families (30-37%) and fall into the range of insect’s whole mitochondrial genomes (13-35%, mean=24%) [32]. Since most of the synonymous sites are at the third codon position, such a bias in G+C content may affect the estimation of evolutionary rates of synonymous and non-synonymous substitutions of the *usp* gene. We therefore tested whether the increase of substitution rate observed in Mecopterida *usp* could be due, at least in part, to GC-biased gene conversion along branch A. First, the two ancestral sequences for the internal nodes at each extremity of branch A were determined by marginal reconstruction performed with Codeml within PAML software (Yang et al. 1997). Then, we identified the predicted substitutions along this branch by comparing these two ancestral sequences. We could not detect any bias in favour of AT→GC substitutions (p-value = 0.4). This means that the current bias in the G+C content of *usp* gene was probably acquired later during evolution. It is therefore unlikely that a phase of GC-biased gene conversion had a significant effect in the global increase of evolutionary rates observed in *usp* during the emergence of Mecopterida.

**Table 3:** **Average percentage of G+C content in codons of *usp***

	**Diptera**	**Lepidoptera**	***Tribolium***	**Blattaria**
Codons	346	398	283	279
First position	57,5%	59,0%	49,7%	57,9%
Second position	38,7%	42,2%	35,3%	40,4%
Third position	**67,5%**	**72,8%**	52,7%	48,9%
All sites	54,6%	58,0%	45,9%	49,1%

## Discussion

The ecdysone receptor (ECR-USP) has undergone a dramatic phase of mutations during the emergence of the Mecopterida superorder [4-6]. This acceleration of evolutionary rate is correlated with structural and functional changes of the LBP of USP and of the heterodimer interface between ECR and USP [7,8]. These results suggested that the maintenance of the ecdysone pathway was achieved, at least in part, through molecular adaptation of the USP LBD. We thus tested the possibility that this domain was under positive selection during the emergence of Mecopterida.

### Constraints and relaxation on USP LBD

Overall, USP is highly constrained. The majority of the sites are under negative selection on all branches. Similar conclusions have been obtained with other nuclear receptors, including RXR, the vertebrate homolog of USP [33-35]. This result is compatible with the idea that USP could be a hub protein. Indeed, proteins with a large number of interactions are usually under purifying selection because any substitution may have pleiotropic effects. At the same time, adaptations are allowed by relaxation in the interactive surfaces that establish transient interactions between protein domains [36]. In the case of the LBD of USP, we detected a relaxation of negative selection within the Mecopterida, relative to non-Mecopterida insects. Most of the relaxed sites, which are on the external surfaces of the protein, may be involved in protein-protein interactions between USP and its coregulators. It is therefore possible that the evolutionary plasticity observed on the surface of the Mecopterida USP LBD reflects adaptability to its different partners. Unfortunately, the network of protein-protein interactions is largely unknown for USP and experimental data are needed to go beyond these speculations. In fact, the complexity of this matter concern all nuclear receptors, for which the analysis of protein interactions are largely restricted to the dimerisation interface and to the small coregulatory groove [37]. The use of original evolutionary methods can yield a better understanding of these interactive sites. For instance, difference evolutionary trace analysis has revealed a new functional site for steroid receptor located on a surface opposite to currently known protein-protein interaction [38].

### Regions of USP LBD under positive selection during Mecopterida emergence

Our previous results suggested that the main functional shifts affecting USP LBD of insects occurred during the emergence of Mecopterida, approximately 280–300 million years ago (early Permian). We therefore used branch-site models to detect changes in selective constraints along this branch. Importantly, we also analysed two more recent branches within the Mecopterida clade in order to decipher the successive state of evolutionary forces acting on USP LBD. Indeed, the interpretation of changes in evolutionary rates must take into account all the biological complexity of the protein, such as the dynamic of the process or the distinction between functional modules. Using this approach, we were able to detect positive selection at amino acids located on the LBP and on the heterodimerisation interface of USP LBD.

The episode of positive selection is correlated with the acquisition of a large LBP filled by fatty acids. In the basal insect *Locusta migratoria* (Orthoptera), USP is able to bind 9-cis retinoic acid, like homologous vertebrate RXR, which natural ligands are fatty acids [39]. In *Bemisia* (Hemiptera) and *Tribolium* (Coleoptera), USP is unable to be activated by RXR ligands and acts as a constitutively silent partner of ECR. In fact, in these species, residues of the loops L6 and L11 fill the deep unliganded pocket and, therefore, USP lacks a proper LBP [7,28]. All the four residues that underwent strong positive selection during the emergence of Mecopterida are located at the bottom of the deep pocket, which is similar to the LBP of RXR. These residues make contacts with the tails of the phospholipid in Mecopterida structures. Later during the diversification of Mecopterida, the same positions became under purifying selection. These results suggest that the Mecopterida pocket was positively selected for a new function, which then became highly constrained and remained largely unchanged until now. What was this innovation? We can hypothesize that the presence of phospholipids allowed the stabilisation of the new Mecopterida LBD. Indeed, this structure is characterised by the acquisition of a new loop L1-3 that locks the LBD in a conformation similar to the one seen for antagonist-bound nuclear receptors [25,26,29]. In that perspective, phospholipids would be analogous to pharmacological chaperone, which are small molecules that help to maintain the correct folding of mutant proteins [40]. This interpretation is in agreement with a previous proposition, based on an information-theoretic approach, according to which the ligand-contacting residues perform a dual role: a functional role in ligand recognition and a structural role as core residues [41]. Thus, the structure of the LBP of USP was highly plastic during insect evolution, adopting three of the four states that are known for nuclear receptors: nutritional sensor (basal insects), real orphan (Hemiptera, Coleoptera) and receptor with a constitutive activity (Mecopterida). The first evolutionary transition involved a loss of function, from a sensor to an orphan receptor [8]. Our results highlight the second transition, from an orphan receptor to a constitutive receptor, which could be seen as a gain of function.

We also detected positive selection in the dimerisation interface during the emergence of Mecopterida. This episode of positive selection is correlated with a loss of ability to homodimerise for both ECR and USP. Non-Mecopterida ECRs are able to homodimerise and are active without USP [42-44]. By contrast, even in presence of their ligand, the ECR LBD monomers of *Heliothis* and *Drosophila* are very unstable in solution and need USP for solubilisation and stabilisation [45,46]. Similarly, non-Mecopterida USPs are able to form homodimers, whereas Mecopterida USPs are not able to do so [7,42,47,48]. It seems therefore that Mecopterida USP acts as a chaperone-like partner for ECR stabilisation. We have shown previously that, during the emergence of Mecopterida, a relaxation of evolutionary constraints occurred at a site that affects homodimerisation but not heterodimerisation [8,49]. This residue is the glutamine Gln457 in helix H10, for which we provide here evidence for strong positive selection on the branch leading to Mecopterida (Figure 5A). This result confirms our first hypothesis proposing that substitution of this site located in the core of the dimerisation surface may have induced a loss of homodimerisation for USP. Positive selection was found at those three sites in an independent study [50].

Importantly, if the homodimerisation ability has been lost in Mecopterida, on the contrary, the heterodimerisation between ECR and USP has been conserved by all arthropods and was even reinforced in Mecopterida. How can we explain the maintenance of the functional interaction between ECR and USP, despite the challenging burst of mutations that occurred in the stem lineage of Mecopterida? The heterodimerisation between ECR and USP depends largely on a core of residues localised in helices H9 and H10 of the LBDs. While the sequence of this interface is well conserved in all insects, in Mecopterida, the loop L8-9 of USP interacts with the helix H7 of ECR, creating a symmetric and larger heterodimerisation surface [8]. This novel surface is a consequence of the Mecopterida specific structural divergences of USP, where the loop L1-3 and the presence of a phospholipid, which is in tight contact with L1-3 and H3, induce a 15°-torsion of a sub-domain of USP LBD towards ECR. If the maintenance of heterodimerisation depends on this new surface, then positive selection should be detected at this specific enlargement on the stem lineage of Mecopterida. We have confirmed this prediction for two sites of USP: Arg426 in helix H9 and Arg399 in helix H7 (Figure 5A). The arginine Arg426 is involved in the Mecopterida specific enlargement of the heterodimerisation surface, while the Arg399 was predicted to form an electrostatic interaction with ECR in the ancestral Mecopterida model [8]. In addition, we detected positive selection at the site Asp452, which is located in the core interface and makes contacts with two residues of the helix H9 of ECR. It is thus possible that this substitution played a role in the reinforcement of the ECR-USP heterodimerisation. If we assume that the reinforcement of ECR-USP heterodimerisation is a selectively favoured phenotype, then every amino acid replacement causing a slight increase in interaction would be selectively favoured. However, once binding is optimum, no further changes will be favoured. Thus, a phase of purifying selection would follow an initial phase of positive selection. We have confirmed this expectation by showing that the same four positions described above became under purifying selection during the diversification of Mecopterida. In conclusion, the dimerisation interface of USP has experienced modifications of selective constraints during the emergence of Mecopterida, followed by a phase of constrained evolution.

Although our results suggested that coevolution has maintained the interaction between ECR and USP, we could not detect any significant modification of evolutionary constraints in the dimerisation surface of ECR [8]. Recent random-sites model analyses confirmed the absence of positive selection in ECR [50]. It seems therefore that the helix H7 of ECR, which is implicated in the new Mecopterida interface with USP, was able to adapt to its changing partner in a manner independent of selection. In order to explain the evolution of this domain, it may be necessary to explore the role of molecular drive, which includes all the DNA turnover mechanisms that change the genetic composition of a population, independently of selection and drift [51]. There is indeed a renewed interest in the evolutionary forces, other than natural selection or drift, that can induce co-evolution between functionally interacting genetic systems in order to maintain essential functions [15,52]. However, interestingly, six residues with elevated ω were identified in the core interface of Mecopterida ECR, suggesting that the dimerisation core of ECR, like that of USP/RXR, may also be under relaxed constraint in the Mecopterida clade [50].

Within the family of nuclear receptors, evidence for positive selection has been reported in a few other cases, such as PXR (NR1I2), CAR (NR1I3) and ERα (NR3A1) [33,53,54]. For example, positive selection was detected at amino acids involved in ligand binding of the frog PXRs, which have lost broad specificity for ligands and gained efficient activation by endogenous amphibian-specific benzoates [55]. A recent study has shown that RXR, the USP homolog, was under positive selection immediately following the first round (1R) and second round (2R) of whole genome duplications in vertebrates [34]. Most of the positive selected sites were located either in the LBD or in the N-terminal region, which contain activation domains (AF-1 and AF-2) and phosphorylation sites that control RXR expression. During this evolutionary transition, it seems that positive selection did not affect the LBP or the dimerisation interface of RXR (except for one site of helix H9 on the post-duplication branch leading to RXRγ). The authors thus conclude: “The presence of positive selection/accelerated rates in the paralogs, coupled with the evidences of altered expression of paralogous genes explains the preservation of the additional RXR copies following genome duplication” [34]. It would be interesting to analyse the evolution of the N-terminal region of USP and ECR, but the structure and the function of these highly divergent domains are largely unknown in insects.

### Episodic evolution of USP

In conclusion, our results allow to define three phases of evolution for the LBD of USP in Mecopterida. We can now propose a model to explain this episodic mode of evolution. We have previously proposed that the ancestral state of USP LBD was characterised by the absence of a LBP and the ability to form homodimers as well as heterodimers with ECR [7,8]. During the emergence of Mecopterida, an unexplained acceleration of evolutionary rate was probably responsible for the insertion of 11 residues in the loop L1-3. This insertion is the primordial event in our hypothesis, because it changed the structure of the LBD in two ways. First, it increased the size of the heterodimer interface. Second, it restored a LBP. However, this new pocket is very large and filled with phospholipids, which is unusual for a nuclear receptor of the NR2B group. These modifications allowed the exploration of new functions, as revealed by the detection of positive selection at these two regions during the first phase of Mecopterida evolution. The new LBP probably allowed the maintenance of a correct folding for the whole LBD, while the new extended surface of dimerisation was associated with a reinforcement of the obligatory partnership between ECR and USP, at the expense of homodimerisation. Therefore, the correlation between positive selection and functional innovations suggests that the USP LBD underwent molecular adaptation during the emergence of Mecopterida. One limit of this kind of analysis is the difficulty to distinguish between positive selection and relaxation of negative selection [11]. However, the availability of crystal structures and functional data provide *a priori* biological hypotheses regarding the amino acids upon which positive selection is expected. Once the new functions are effective, no further changes will be favoured. Thus, the initial phase of positive selection should be followed by a second phase of purifying selection. In agreement with this hypothesis, we found that the new functional domains were under strong negative selection during the subdivision of Mecopterida between Amphiesmenoptera and Antliophora. This second phase of evolution explains the conservation of the new surfaces among Mecopterida species. Finally, we could characterise a third phase of evolution during the subdivision of Diptera into Brachycera and Nematocera. On this branch, USP LBD evolved mainly under a relaxation of negative selection, with neutrality at some sites. However, the new Mecopterida functional domains remained highly constrained (except the tyrosine Y372 in the LBP of Diptera). The alternation of selective and conservative periods during the transformation of USP LBD in insects provides an example of the episodic model of protein evolution, a major mechanism recently revealed by application of new methods at the genomic level [12,56,57].

## Conclusions

The function of the heterodimer ECR-USP in the ecdysone response is conserved in all insects, including Mecopterida [58]. Therefore, although we cannot exclude the possibility that other biological functions have changed with USP, it seems that adaptation of this protein allowed maintenance rather than innovation. It is an important property of living organisms to remain constant in the face of change. The concept of canalisation describes the capacity of a population to produce the same phenotype despite genetic or environmental variability. At the molecular level, the capacity of a protein to restore functionality after a mutational event could be qualified as “molecular resilience”. In the case of USP, the insertion in the loop L1-3 was likely the initial “trauma”, induced by a phase of increased mutation rate at the origin of Mecopterida. Resilience was probably dependent on the existence of pre-existing functionally neutral mutations that accumulated in the LBD of USP. Once the insertion occurred, only a specific combination of these permissive mutations became adaptive, because they could compensate for the structural changes induced by the insertion. Epistatic contacts between and within proteins are essential to allow a genetic network to assimilate new genotypes while remaining functional, because any loss of function could be deleterious [1,59]. Like canalisation, molecular resilience would therefore allow evolvability by accumulation of genetic diversity without modification of the phenotype. In that perspective, the molecular adaptation of USP could be seen as a process of resilience for the ecdysone receptor.

## Methods

### Insects

Drosophilidae species from the Gif laboratory were obtained from different origins: *Drosophila simulans* (La Réunion, France), *Drosophila mauritiana* (Mauritius), *Drosophila santomea* (Sao Tomé), *Drosophila teissieri* (Congo), *Drosophila malerkotliana* (Madagascar), *Drosophila (Zaprionus) tuberculatus* (Ivory Coast), *Drosophila limbata* (France), *Drosophila caribiana* (Martinique, France), *Drosophila funebris* (France).

The species of *Tribolium* were reared on wheat flour with 5% of dried yeast extract at room temperature. The USDA-ARS Grain Marketing and Production Research Centre (Manhattan, Kansas) provided all the species, except *Tribolium destructor*, which was provided by Durdica Ugarkovic (Ruder Bosković Institute, Zagreb, Croatia).

Concerning Blattaria species, *Blattella germanica* was from the strain maintained in the Institute of Evolutionary Biology (CSIC-UPF), in Barcelona, whereas *Blatta orientalis*, *Periplaneta americana*, *Periplaneta japonica*, *Periplaneta australasiae*, *Periplaneta brunnea* and *Periplaneta fuliginosa*, were kindly provided by Pierre Deleporte (Station Biologique de Paimpont, Université de Rennes). Blattarian specimens were maintained in the dark at 30 ±1°C and 60-70% RH prior to RNA extraction.

### Cloning and sequencing

We isolated 43 new sequences using PCR with degenerated primers. Most of the partial sequences for USP encompass regions from the beginning of the DNA binding domain (zinc-finger 1 or zinc-finger 2) to the last helix (H12) of the LBD. Sequences of the mitochondrial cytochrome oxidase subunit 1 (COX1, used as a reference) are almost complete, including the twelve membrane-spanning helices. Available sequences were obtained from GenBank. All accession numbers are shown on Additional file 1: Table S1.

Genomic DNAs of Drosophilidae were prepared as described previously [60] from either live stocks, or frozen or ethanol-preserved flies from the Gif LEGS laboratory. Partial *usp* (intronless in *Drosophila*) and *cox1* sequences were amplified by PCR using a set of primers listed in Additional file 10: Table S7. For *usp*, two rounds of nested amplifications were performed with Amplitaq Gold (Applied Biosystems) in a Trioblock device (Biometra) as follows: first round: primers U50dbis/U31dbis; 45 cycles; annealing temperature 60°C; elongation for 1mn45 at 72°C; second round: primers U51dbis/U33dbis; annealing temperature 60°C; elongation for 1mn30 at 72°C. Amplicons of the correct size were purified and cloned into the pGEM-T easy vector (Promega). For *cox1*, samples were amplified with primer COIDIR (forward) combined with various reverse primers using the following conditions: 45 cycles, annealing temperature 50°C; elongation for 1mn30 at 72°C. Purified amplicons were sequenced directly without cloning. For some species, the data were completed with predicted sequences provided by the “*Drosophila* 12 Genomes Consortium” [61].

Sequences for *Tribolium* USP were obtained exactly as described previously [5], using RT-PCR with degenerated primers. Sequences for cytochrome oxidase I (COX1) were obtained by PCR on total DNA. Primers are listed in Additional file 10: Table S7. All combinations of primers were used in semi-nested PCR amplifications in 50 μl volume with 10 mM Tris–HCl pH = 8.3, 50 mM KCl, 1.5 mM MgCl_2_, 0.25mM of each dNTP, 2.5 U AmpliTaq Gold DNA polymerase (Applied Biosystems) and 300 ng of each primer. Reactions were performed in a Biometra T3 Thermocycler, using the following protocol: 2 min at 95°C; cycles 1–5: 94°C 1 min, 45°C 1 min, 72°C 1 min; cycles 6–10: 94°C 1 min, 50°C 1 min, 72°C 1 min; cycles 11–30: 94°C 1 min, 55°C 1 min, 72°C 1 min; followed by terminal elongation for 10 min at 72°C. PCR products were cloned into a TA cloning vector (Invitrogen) and transformed into competent cells according to the manufacturer’s instructions. Sequencing reactions were performed using the ABI PRISM BigDye Terminator v1.1 cycle sequencing kit (Applied Biosystems).

In the case of Blattaria, total RNA was isolated with the GenEluteTM Mammalian Total RNA kit (Sigma) and cDNAs were obtained as described [62]. For RXR amplifications, primers based on the amino acid motif conserved in all RXR/USP sequences located upstream from the DNA-binding domain (DBD) and from the ligand-binding domain (LBD) were used. PCR amplifications were performed using, as a template, cDNA generated by reverse transcription from polyA+ RNA obtained from the different species under study. Amplifications were carried out for 30 cycles at 94°C for 30 s, 52°C for 1 min, 72°C for 1 min, followed by a terminal elongation for 5 min at 72°C. The amplified fragments were sub-cloned into the pSTBlue-1 vector (Novagen) and sequenced. For cytochrome oxidase I (COX1) amplifications, the strategy was the same as before. Primers are listed in Additional file 10: Table S7.

### Sequence dataset and insect’s phylogeny

In order to estimate the site-specific ratio of non-synonymous/synonymous substitutions (*d*_N_/*d*_S_ or *ω*) through whole tree analyses, we used Maximum Likelihood methods and codon substitution models, implemented in the Codeml program from the PAML package [21,63]. Codons of insect sequences of *usp* and *cox1* genes were aligned using the program Muscle implemented in SeaView [64]. Positions with gaps were removed. All accession numbers are supplied in Additional file 1: Table S1. The predefined topology of insects phylogeny, imposed for the ML computations, is shown on Figure 1. This consensus tree is based on classical taxonomic data, as well as specific references concerning the following groups: Diptera [65], Drosophilidae [16,66], Lepidoptera [67,68], Tenebrionidae [17], Blattaria [18,19,69] and insects [70-72]. We carried out statistical analyses with the R software [73].

### Detection of selective constraints

In a first set of analyses, we characterized the pattern of site-specific *d*_N_/*d*_S_ ratios along *usp* sequences within two non-Mecopterida (5 Tenebrionidae, 7 Blattaria) and three Mecopterida groups (16 Drosophilidae, non-Drosophilidae 13 Diptera and 6 Lepidoptera), based on the alignment of 283, 279, 377, 346 and 398 codons, respectively. In order to detect positive selection within these groups, we compared the likelihood of two pairs of site models: M1a/M2a and M7/M8 [22,74]. The null model M1a (nearly neutral) assumes two classes of sites, with either *ω*_*0*_ ≤ 1 (negative or purifying selection) or *ω*_*1*_ = 1 (neutral). The alternative model M2a (selection) adds a third class of sites with *ω*_*2*_ ≥ 1 (positive selection). The null model M7 assumes that *ω* ratios vary among codons, following a β distribution in the interval 0<*ω*<1. The alternative model M8 adds an extra class of sites under neutral evolution or positive selection with *ω*_*S*_ ≥ 1. We fixed ten categories of sites for the discrete approximate of the β distribution. This high number of categories allows to use the Bayesian approach to calculate the posterior probabilities for site classes and expected site-specific *d*_N_/*d*_S_ values [74]. The best codon model for each group was run ten times, using different initial values for parameters, in order to test for convergence during the process of likelihood maximization by the Codeml algorithm. β distributions were compared by Kolmogorov-Smirnov tests. The average evolutionary rate of USP protein was compared between Drosophilidae, Tenebrionidae and Blattaria by using mean *d*_N_ of COX1 as a reference [20]. Site-specific *d*_N_/*d*_S_ values estimated along the *usp* sequence were projected onto the 3D protein structure of the USP LBD domain of *Drosophila* (1HG4), *Heliothis* (1R1K), or *Tribolium* (2NXX). Because saturation was detected among Diptera *usp*, we assessed the accuracy of the estimate of the β distribution function for this clade. For this, we re-estimated the β distribution parameters with the same Codeml analysis (under model M7) from 50 simulated alignments, which were generated with the Evolver program of the PAML package using the global parameters established for the real *usp* Diptera dataset (number of sequences, sequence length, topology and branch lengths of the tree, β distribution function for *d*_N_/*d*_S_, Ts/Tv ratio, codon usage) (Additional file 4: Figure S1).

In a second type of analyses performed with branch-site models from the Codeml program, we defined three sets of *usp* sequences in order to detect site-specific changes in selective constraints along three internal branches that are relevant to the early history of Mecopterida: branch A is the stem lineage of Mecopterida, branch B delineates the two subgroups of Mecopterida (Amphiesmenoptera: Lepidoptera, Trichoptera and Antliophora: Diptera, Mecoptera, Siphonaptera), and branch C follows the subdivision of Diptera into Brachycera and Nematocera (Figure 1). We applied tests 1 and 2 from Zhang *et al.*[30] to assess whether relaxation or positive selection occurred along these branches. For this, the likelihood of three models were computed: the nearly neutral model (M1a), assuming constrained and neutral residues with no shifts along the tested branch; the modified branch-site model A, allowing some sites to undergo a *ω*_2_>1 for the predefined branch, thus considering possible positive selection; a third branch model with site-specific relaxation built in the same way as the modified branch-site model A but with *ω*_*2*_ = 1 fixed. As recommended by Zhang *et al.*[30], we considered χ^2^ distributions with two and one degree(s) of freedom for the null distribution of the likelihood ratio tests 1 and 2 (respectively), which guarantees a conservative test of positive selection (test 2). Although tests 1 and 2 are claimed to be robust to the large evolutionary distances [21], we controlled false positive rate with a simulation methodology as previously proposed by Studer *et al.*[13]. For this, tests 1 and 2 were applied on 100 alignments generated with Evolver under the null model of the test (M1a and modified branch-site model A with *ω*_*2*_ = 1) using the global parameters established for the real dataset (branch A, B, or C). Lastly, we identified sites that experienced a selective shift along the three branches of interest. To this end, we used the Bayesian procedure implemented in Codeml to calculate the posterior probability that a given site is under relaxation or under positive selection [74]. We projected these probabilities onto the 3D structures of USP DBD (2HAN) and LBD domains of *Drosophila* (1HG4).

## Abbreviations

USP: Ultraspiracle; ECR: Ecdysone receptor; LBD: Ligand Binding Domain; LBP: Ligand Binding Pocket; DBD: DNA Binding Domain.

## Competing interests

The authors declare that they have no competing interests.

## Authors' contributions

AC, Fbo and VL designed the study. JLDL, OM, DM and FBo performed the cloning and sequencing experiments. AC carried out the detection of selective constraints. FBr and FBo participated in the analysis of sequences. TI participated in the analysis of 3D structures. AC and FBo analyzed the data, interpreted the results and wrote the manuscript. JLDL, TI, XB and VL critically revised the manuscript. All authors read and approved the final manuscript.

## Supplementary Material

Additional file 1 **Table S1.** Accession numbers of sequences used for alignments. Click here for file

Additional file 2 **Table S3.** dN/dS of usp in Drosophilidae, Diptera, Lepidoptera, Tenebrionidae and Blattaria. Click here for file

Additional file 3 **Table S4.** Standard errors [SE] associated to the parameters of the models fitted in the likelihood analysis reported in Table 1. Click here for file

Additional file 4 **Figure S1.** Robustness of dN/dS estimates to saturation in Diptera usp dataset. The accuracy of the estimate of the β distribution function was tested by re-performing the Codeml analysis (under model M7) on 50 simulated alignments, which were generated with Evolver in the PAML package using the global parameters established for the real usp Diptera dataset (number of sequences, sequence length, topology and branch lengths of the tree, β distribution function for dN/dS, Ts/Tv ratio, codon usage). Bold lines are the same as in Figure 2: Drosophilidae: red; Diptera: purple; Lepidoptera: orange; Tenebrionidae: green; Blattaria: blue. A thin pink line was added for each ones of the 50 re-analysis of the simulated alignments. Click here for file

Additional file 5 **Table S5.** Comparison between usp and cox1 substitution rates. Click here for file

Additional file 6 **Figure S2.** Evolutionary rates on the surface of USP structures in four extant groups of insects. Site-specific dN/dS were projected onto the crystal structure of the USP LBD domain of Tribolium (2NXX, ECR-USP) for Blattaria (A) and Tenebrionidae (B), of Drosophila (1HG4, USP) for Diptera (C) and of Heliothis (1R1K, ECR-USP) for Lepidoptera (D). The values are distributed along a colour scale from blue (low dN/dS) to red (high dN/dS). Sequences not available for the estimation of evolutionary rates are in white. Views on the left show the side of the LBD that is near ECR, while views on the right show the same structure after a rotation of 180°. Click here for file

Additional file 7 **Table S6.** Posterior probabilities for each site to belong to the site-class under positive selection (along branch A or branch B) or relaxation (along branch C). Click here for file

Additional file 8 **Figure S3.** Selection in the putative coactivation surface during the radiation of Mecopterida. Posterior probabilities for each site to belong to the site-class under positive selection (along branch A or branch B) or relaxed evolution (along branch C) were projected onto the crystal structure of the USP LBD domain of Drosophila (1HG4). Probabilities are distributed along a colour scale from yellow (p=0) to red (p=1). Sequences not available for the estimation of evolutionary rates are in white. (A) Branch A, stem lineage of Mecopterida. (B) Branch B, subdivision between Amphiesmenoptera (Lepidoptera, Trichoptera) and Antliophora (Diptera, Mecoptera, Siphonaptera). (C) Branch C, subdivision of Diptera into the suborders Brachycera and Nematocera. (D) The coactivation surface, defined by homology with RXR, is shown in pink. The helix H12 is showed in green. Click here for file

Additional file 9 **Table S2.** Relative synonymous codon usage (RSCU). Click here for file

Additional file 10 **Table S7.** Degenerated primers used to clone usp and cox1 genes. Click here for file
