# Supplementary material for: Molecular adaptation and resilience of the insect’s nuclear receptor USP
Source: BMC Evol Biol. 2012 Oct 5;12:199. doi: 10.1186/1471-2148-12-199 (PMC3520820; doi:10.1186/1471-2148-12-199)
Supplement: Additional file 1 — Table S1. Accession numbers of sequences used for alignments. [file 1471-2148-12-199-S1.doc]

| **Group** |  | ***Species*** | **USP** | **COX1** |
| --- | --- | --- | --- | --- |
| Diptera | Drosophilidae | *Drosophila melanogaster* | X53417 | NC_001709 ^*^ |
|  |  | *Drosophila sechellia* | M_002040402 ^*^ | NC_005780 ^*^ |
|  |  | *Drosophila simulans* | JQ679105 ^#^ | NC_005781 ^*^ |
|  |  | *Drosophila mauritiana* | JQ679106 ^#^ | NC_005779 ^*^ |
|  |  | *Drosophila yakuba* | XM_002100290 ^*^ | NC_001322 ^*^ |
|  |  | *Drosophila santomea* | JQ679108 ^#^ | JQ679120 ^#^ |
|  |  | *Drosophila teissieri* | JQ679107 ^#^ | JQ679119 ^#^ |
|  |  | *Drosophila erecta* | XM_001982547 ^*^ | JQ679121 ^#^ |
|  |  | *Drosophila ananassae* | XM_001966427 ^*^ | JQ679117 ^#^ |
|  |  | *Drosophila malerkotliana* | JQ679109 ^#^ | JQ679118 ^#^ |
|  |  | *Drosophila pseudoobscura* | XM_001355316 ^*^ | JQ679122 ^#^ |
|  |  | *Drosophila willistoni* | XM_002071308 ^*^ | JQ679116 ^#^ |
|  |  | *Zaprionus tuberculatus* | JQ679102 ^#^ | JQ679110 ^#^ |
|  |  | *Drosophila hydei* | JQ679104 ^#^ | JQ679112 ^#^ |
|  |  | *Drosophila virilis* | XM_002057324 ^*^ | JQ679111 ^#^ |
|  |  | *Drosophila grimshawi* | XM_001992041 ^*^ | BK006341 ^*^ |
|  |  | *Drosophila limbata* | JQ679101 ^#^ | JQ679113 ^#^ |
|  |  | *Drosophila caribiana* | JQ679103 ^#^ | JQ679114 ^#^ |
|  |  | *Drosophila funebris* | JQ679100 ^#^ | M57908 ^*^ |
|  |  | *Lucilia cuprina* | AAG01569 ^§^ |  |
|  |  | *Aedes aegypti* | AAG24886 ^§^ |  |
|  |  | *Aedes albopictus* | AAF19033 ^§^ |  |
|  |  | *Anopheles gambiae* | XP_320944 ^§^ |  |
|  |  | *Chironomus tentans* | AAC03056 ^§^ |  |
| Siphonaptera |  | *Ctenocephalides felis* | AAV24974 ^§^ |  |
| Lepidoptera |  | *Bombyx mori* | AAC13750 ^§^ |  |
|  |  | *Manduca sexta* | P54779 ^§^ |  |
|  |  | *Heliothis virescens* | CAD28568 ^§^ |  |
|  |  | *Chilo suppressalis* | BAC53670 ^§^ |  |
|  |  | *Plodia interpunctella* | AAT44330 ^§^ |  |
|  |  | *Choristoneura fumiferana* | AAC31795 ^§^ |  |
| Trichoptera |  | *Chimarra marginata* | AAZ38141 ^§^ |  |
| Coleoptera | *Tenebrionidae* | *Tribolium castaneum* | NM_001114294 | AJ312413 ^*^ |
|  |  | *Tribolium freemani* | FJ743717 ^#^ | FJ743724 ^#^ |
|  |  | *Tribolium madens* | FJ743718 ^#^ | FJ743721 ^#^ |
|  |  | *Tribolium destructor* | FJ743719 ^#^ | FJ743723 ^#^ |
|  |  | *Tribolium confusum* | FJ743720 ^#^ | FJ743725 ^#^ |
|  |  | *Tribolium brevicornis* | FJ743716 ^#^ | FJ743722 ^#^ |
|  |  | *Tenebrio molitor* | AJ251542 | *c* |
|  |  | *Leptinotarsa decemlineata* | BAD99298 ^§^ |  |
| Strepsiptera |  | *Xenos pecki* | AAX37291 ^§^ |  |
|  |  | *Xenos vesparum* | AAZ38142 ^§^ |  |
| Hymenoptera |  | *Apis mellifera* | AAF73057 ^§^ |  |
|  |  | *Melipona scutellaris* | AAW02952 ^§^ |  |
| Hemiptera |  | *Bemisia tabaci* | 1Z5XU ^§^ |  |
| Orthoptera |  | *Locusta migratoria* | AAF00981 ^§^ |  |
| Blattaria |  | *Periplaneta americana* | AJ621779 ^#^ | AM114927 ^#^ |
|  |  | *Periplaneta brunnea* | AJ621781 ^#^ | AM114930 ^#^ |
|  |  | *Blatta orientalis* | AJ621784 ^#^ | AM114926 ^#^ |
|  |  | *Periplaneta australasiae* | AJ621780 ^#^ | AM114928 ^#^ |
|  |  | *Periplaneta fuliginosa* | AJ621782 ^#^ | AB126004 ^*^ |
|  |  | *Periplaneta japonica* | AJ621783 ^#^ | AM114929 ^#^ |
|  |  | *Blattella germanica* | AJ854489 | S72627 |
| Crustacea |  | *Uca pugilator* | AAC32789 ^§^ |  |
| Chelicerata |  | *Amblyomma americanum* | AAC15588 ^§^ |  |

**Table S1.** Accession numbers of sequences used for alignments. * genome sequence. # this study. *c*: consensus of: X88966, AJ438073, AJ438074, AJ438095, AJ438096 and EU048284. § Protein accession number of USP sequences used for the reference alignment.
