# Supplementary material for: Molecular adaptation and resilience of the insect’s nuclear receptor USP
Source: BMC Evol Biol. 2012 Oct 5;12:199. doi: 10.1186/1471-2148-12-199 (PMC3520820; doi:10.1186/1471-2148-12-199)
Supplement: Additional file 2 — Table S3. dN/dS of usp in Drosophilidae, Diptera, Lepidoptera, Tenebrionidae and Blattaria. [file 1471-2148-12-199-S2.pdf]

Drosophilidae			Diptera			Lepidoptera			Tenebrionidae			Blattidae		
dmel		M7 dN/dS	dmel		M8 dN/dS	hvir		M7 dN/dS	tcas		M7 dN/dS	bger		M8 dN/dS
						1	L	0,0						
						2	N	0,0						
						3	L	0,1						
						4	E	0,0						
						5	G	0,3						
						6	G	0,3						
						7	F	0,0						
						8	M	0,0						
						9	S	0,0						
						10	P	0,0						
						11	M	0,0						
						12	S	0,0						
						13	P	0,0						
						14	P	0,0						
						15	E	0,0						
						16	M	0,0						
						17	K	0,0						
						18	P	0,0						
						19	D	0,0						
						20	T	0,0						
						21	A	0,0						
						22	M	0,0						
						23	L	0,0						
						24	D	0,0						
						25	G	0,1						
						26	L	0,1						
						27	R	0,0						
						28	D	0,0						
						29	D	0,0						
						30	S	0,2						
						31	T	0,0						
1	S	0,0				32	P	0,2						
2	P	0,0				33	P	0,0						
3	K	0,0				34	P	0,0						
4	A	0,0				35	A	0,3						
5	E	0,0				36	F	0,0						
						37	K	0,0						
						38	N	0,0						
6	S	0,1												
7	P	0,0												
8	V	0,1												
9	P	0,0												
10	F	0,0												
11	M	0,1												
12	Q	0,0												
13	A	0,2												
14	M	0,0												
15	S	0,1												
16	M	0,0												
17	V	0,1												
18	H	0,1												
19	V	0,0												
20	Y	0,0	1	Y	0,0	39	Y	0,0						
21	P	0,0	2	P	0,0	40	P	0,0						
22	P	0,0	3	P	0,0	41	P	0,0						
23	N	0,0	4	N	0,0	42	N	0,0						
24	H	0,0	5	H	0,0	43	H	0,0						
25	P	0,0	6	P	0,0	44	P	0,0				1	P	0,0
26	L	0,0	7	L	0,0	45	L	0,0				2	L	0,0
27	S	0,0	8	S	0,0	46	S	0,0				3	S	0,0
28	G	0,1	9	G	0,0	47	G	0,0				4	G	0,0
29	S	0,1	10	S	0,1	48	S	0,0				5	S	0,0
30	K	0,0	11	K	0,0	49	K	0,0				6	K	0,0
31	H	0,0	12	H	0,0	50	H	0,0				7	H	0,0
32	L	0,0	13	L	0,0	51	L	0,0				8	L	0,0
33	C	0,0	14	C	0,0	52	C	0,0				9	C	0,0

34	S	0,0	15	S	0,0	53	S	0,0				10	S	0,0	
35	I	0,0	16	I	0,0	54	I	0,0				11	I	0,0	
36	C	0,0	17	C	0,0	55	C	0,0				12	C	0,0	
37	G	0,0	18	G	0,0	56	G	0,0				13	G	0,0	
38	D	0,0	19	D	0,0	57	D	0,0				14	D	0,0	
39	R	0,0	20	R	0,0	58	R	0,0				15	R	0,0	
40	A	0,0	21	A	0,0	59	A	0,0				16	A	0,0	
41	S	0,0	22	S	0,0	60	S	0,0				17	S	0,0	
42	G	0,0	23	G	0,0	61	G	0,0				18	G	0,0	
43	K	0,0	24	K	0,0	62	K	0,0				19	K	0,0	DBD Zinc-finger 1
44	H	0,0	25	H	0,0	63	H	0,0				20	H	0,0	
45	Y	0,0	26	Y	0,0	64	Y	0,0				21	Y	0,0	
46	G	0,0	27	G	0,0	65	G	0,0				22	G	0,0	
47	V	0,0	28	V	0,0	66	V	0,0				23	V	0,0	
48	Y	0,0	29	Y	0,0	67	Y	0,0				24	Y	0,0	
49	S	0,0	30	S	0,0	68	S	0,0				25	S	0,0	
50	C	0,0	31	C	0,0	69	C	0,0				26	C	0,0	
51	E	0,0	32	E	0,0	70	E	0,0				27	E	0,0	
52	G	0,0	33	G	0,0	71	G	0,0				28	G	0,0	
53	C	0,0	34	C	0,0	72	C	0,0				29	C	0,0	
54	K	0,0	35	K	0,0	73	K	0,0				30	K	0,0	
55	G	0,0	36	G	0,0	74	G	0,0				31	G	0,0	
56	F	0,0	37	F	0,0	75	F	0,0				32	F	0,0	
57	F	0,0	38	F	0,0	76	F	0,0				33	F	0,0	
58	K	0,0	39	K	0,0	77	K	0,0				34	K	0,0	
59	R	0,0	40	R	0,0	78	R	0,0	1	R	0,0	35	R	0,0	
60	T	0,0	41	T	0,0	79	T	0,0	2	T	0,0	36	T	0,0	
61	V	0,0	42	V	0,0	80	V	0,0	3	V	0,0	37	V	0,0	
62	R	0,0	43	R	0,0	81	R	0,0	4	R	0,0	38	R	0,0	
63	K	0,0	44	K	0,0	82	K	0,0	5	K	0,0	39	K	0,0	
64	D	0,0	45	D	0,0	83	D	0,0	6	D	0,0	40	D	0,0	
65	L	0,0	46	L	0,0	84	L	0,0	7	L	0,0	41	L	0,0	
66	T	0,1	47	T	0,0	85	T	0,2	8	S	0,0	42	S	0,0	
67	Y	0,0	48	Y	0,0	86	Y	0,0	9	Y	0,0	43	Y	0,0	
68	A	0,0	49	A	0,0	87	A	0,0	10	A	0,0	44	A	0,0	
69	C	0,0	50	C	0,0	88	C	0,0	11	C	0,0	45	C	0,0	
70	R	0,0	51	R	0,0	89	R	0,0	12	R	0,0	46	R	0,0	
71	E	0,0	52	E	0,0	90	E	0,0	13	E	0,0	47	E	0,0	
72	N	0,0	53	N	0,0	91	E	0,1	14	E	0,1	48	D	0,0	
73	R	0,0	54	R	0,0	92	R	0,1	15	K	0,0	49	K	0,0	
74	N	0,0	55	N	0,0	93	N	0,0	16	N	0,0	50	N	0,0	
75	C	0,0	56	C	0,0	94	C	0,0	17	C	0,0	51	C	0,0	
76	I	0,0	57	I	0,1	95	I	0,0	18	I	0,0	52	I	0,0	
77	I	0,0	58	I	0,0	96	I	0,0	19	I	0,0	53	I	0,0	
78	D	0,0	59	D	0,0	97	D	0,0	20	D	0,0	54	D	0,0	DBD Zinc-finger 2
79	K	0,0	60	K	0,0	98	K	0,0	21	K	0,0	55	K	0,0	
80	R	0,0	61	R	0,0	99	R	0,0	22	R	0,0	56	R	0,0	
81	Q	0,0	62	Q	0,0	100	Q	0,0	23	Q	0,0	57	Q	0,0	
82	R	0,0	63	R	0,0	101	R	0,0	24	R	0,0	58	R	0,0	
83	N	0,0	64	N	0,0	102	N	0,0	25	N	0,0	59	N	0,0	
84	R	0,0	65	R	0,0	103	R	0,0	26	R	0,0	60	R	0,0	
85	C	0,0	66	C	0,0	104	C	0,0	27	C	0,0	61	C	0,0	
86	Q	0,0	67	Q	0,0	105	Q	0,0	28	Q	0,0	62	Q	0,0	
87	Y	0,0	68	Y	0,0	106	Y	0,0	29	Y	0,0	63	Y	0,0	
88	C	0,0	69	C	0,0	107	C	0,0	30	C	0,0	64	C	0,0	
89	R	0,0	70	R	0,0	108	R	0,0	31	R	0,0	65	R	0,0	
90	Y	0,0	71	Y	0,0	109	Y	0,0	32	Y	0,0	66	Y	0,0	
91	Q	0,0	72	Q	0,0	110	Q	0,0	33	Q	0,1	67	Q	0,0	
92	K	0,0	73	K	0,0	111	K	0,0	34	K	0,0	68	K	0,0	
93	C	0,0	74	C	0,0	112	C	0,0	35	C	0,0	69	C	0,0	
94	L	0,0	75	L	0,0	113	L	0,0	36	L	0,0	70	L	0,0	
95	T	0,1	76	T	0,1	114	A	0,0	37	N	0,1	71	S	0,0	
96	C	0,0	77	C	0,0	115	C	0,0	38	M	0,0	72	M	0,0	
97	G	0,0	78	G	0,0	116	G	0,0	39	G	0,0	73	G	0,0	
98	M	0,0	79	M	0,0	117	M	0,0	40	M	0,0	74	M	0,0	
99	K	0,0	80	K	0,0	118	K	0,0	41	K	0,0	75	K	0,0	
100	R	0,0	81	R	0,0	119	R	0,0	42	R	0,0	76	R	0,0	
101	E	0,0	82	E	0,0	120	E	0,0	43	E	0,0	77	E	0,0	
102	A	0,0	83	A	0,0	121	A	0,0	44	A	0,0	78	A	0,0	

103	V	0,0	84	V	0,0	122	V	0,0	45	V	0,0	79	V	0,0	
104	Q	0,0	85	Q	0,0	123	Q	0,0	46	Q	0,0	80	Q	0,0	DBD T-box
105	E	0,0	86	E	0,0	124	E	0,0	47	E	0,0	81	E	0,0	
106	E	0,0	87	E	0,0	125	E	0,0	48	E	0,0	82	E	0,0	
107	R	0,0	88	R	0,0	126	R	0,0	49	R	0,0	83	R	0,0	
108	Q	0,0	89	Q	0,0	127	Q	0,0	50	Q	0,0	84	Q	0,0	
109	R	0,0	90	R	0,0	128	R	0,0	51	R	0,0	85	R	0,0	
110	G	0,0	91	G	0,0	129	A	0,2	52	T	0,0	86	T	0,0	
									53	K	0,0	87	K	0,0	
111	A	0,0	92	A	0,1	130	A	0,0	54	D	0,0	88	E	0,0	
112	R	0,0	93	R	0,0	131	R	0,0	55	R	0,0	89	R	0,0	
113	N	0,2													
114	A	0,1													
115	A	0,1													
116	G	0,0													
117	R	0,0													
118	L	0,1													
119	A	0,2													D domain
						132	G	0,1	56	D	0,0	90	D	0,0	
						133	T	0,1	57	T	0,0	91	Q	0,0	
120	D	0,0	94	D	0,0	134	E	0,0	58	S	0,0	92	N	0,0	
121	D	0,1	95	D	0,1	135	D	0,0	59	E	0,0	93	E	0,0	
122	F	0,0	96	F	0,0	136	A	0,1	60	V	0,0	94	V	0,0	
123	M	0,1	97	M	0,1	137	H	0,1	61	E	0,0	95	E	0,0	
124	T	0,1	98	T	0,1	138	P	0,0	62	S	0,0	96	S	0,0	
125	N	0,1	99	N	0,1	139	S	0,0	63	T	0,0	97	T	0,0	
126	S	0,1	100	S	0,1	140	S	0,0	64	S	0,0	98	S	0,0	
127	V	0,0	101	V	0,1	141	S	0,0	65	N	0,0	99	S	0,0	
128	S	0,0	102	S	0,1	142	V	0,0	66	L	0,1	100	L	0,0	
						143	Q	0,0	67	Q	0,0	101	H	0,0	
									68	A	0,0	102	T	0,0	
129	R	0,0	103	R	0,0										
130	D	0,0	104	D	0,0	144	E	0,0	69	D	0,0	103	D	0,0	
131	F	0,1	105	F	0,1	145	L	0,0	70	M	0,0	104	M	0,0	
132	S	0,1	106	S	0,1	146	S	0,0	71	P	0,0	105	P	0,0	
133	I	0,0	107	I	0,0	147	I	0,0	72	L	0,0	106	V	0,0	
134	E	0,0	108	E	0,0	148	E	0,0	73	E	0,0	107	E	0,0	
135	R	0,0	109	R	0,0	149	R	0,0	74	R	0,0	108	R	0,0	
136	I	0,1	110	I	0,1	150	L	0,0	75	I	0,0	109	I	0,0	LBD H1
137	I	0,2	111	I	0,9	151	L	0,2	76	I	0,0	110	L	0,0	
138	E	0,1	112	E	0,1	152	E	0,0	77	E	0,0	111	E	0,0	
139	A	0,0	113	A	0,0	153	M	0,1	78	A	0,0	112	A	0,0	
140	E	0,0	114	E	0,0	154	E	0,0	79	E	0,0	113	E	0,0	
141	Q	0,0	115	Q	0,0				80	K	0,0	114	K	0,0	
142	R	0,0	116	R	0,1				81	R	0,0	115	R	0,0	
143	A	0,0	117	A	0,1				82	V	0,0	116	V	0,0	
144	E	0,0	118	E	0,0				83	E	0,0	117	E	0,0	
145	T	0,2	119	T	0,1				84	C	0,0	118	C	0,0	
146	Q	0,2	120	Q	0,2				85	N	0,0	119	K	0,0	
									86	D	0,0				
						155	S	0,2	87	P	0,0				
						156	L	0,0	88	L	0,0				
						157	V	0,0	89	V	0,0				
						158	A	0,0	90	A	0,0				
												120	S	0,2	
												121	E	0,0	
												122	Q	0,1	
												123	Q	0,0	
												124	V	0,0	
												125	E	0,0	
												126	F	0,0	
												127	E	0,0	
												128	S	0,0	
												129	A	0,0	
						159	D	0,0							
						160	P	0,2							
						161	S	0,3							

						162	E	0,0							
						163	E	0,0							
									91	L	0,0				
									92	V	0,1				
									93	V	0,0				
									94	N	0,0				
									95	E	0,0				
									96	N	0,0				
									97	T	0,0				
									98	T	0,0				
147	C	0,1	121	C	0,0										
148	G	0,0	122	G	0,0										
149	D	0,0	123	D	0,0										
150	R	0,0	124	R	0,1										
151	A	0,0	125	A	0,1										
152	L	0,0	126	L	0,0	164	F	0,2							
153	T	0,2	127	T	0,3	165	Q	0,0							
154	F	0,1	128	F	0,0	166	F	0,1							
155	L	0,0	129	L	0,0	167	L	0,0							
156	R	0,1	130	R	0,0	168	R	0,0							
157	V	0,0	131	V	0,0	169	V	0,0							
158	G	0,0	132	G	0,0	170	G	0,0							
159	P	0,0	133	P	0,1	171	P	0,0							
160	Y	0,1	134	Y	0,1	172	D	0,2							
161	S	0,0	135	S	0,0	173	S	0,0							
162	T	0,1	136	T	0,0	174	N	0,2							
163	V	0,0	137	V	0,0	175	V	0,0							
164	Q	0,0	138	Q	0,0	176	P	0,0							
165	P	0,1	139	P	0,1	177	P	0,1							
166	D	0,0	140	D	0,0	178	K	0,1							
167	Y	0,0	141	Y	0,0	179	F	0,1							
168	K	0,0	142	K	0,0	180	R	0,0							
169	G	0,0	143	G	0,0	181	A	0,0							
170	A	0,0	144	A	0,0	182	P	0,0							
171	V	0,0	145	V	0,0	183	V	0,0	99	V	0,0	130	V	0,0	
172	S	0,0	146	S	0,0	184	S	0,0	100	N	0,0	131	T	0,0	
173	A	0,0	147	A	0,1	185	S	0,2	101	N	0,0	132	N	0,0	
174	L	0,0	148	L	0,0	186	L	0,0	102	I	0,0	133	I	0,0	
175	C	0,0	149	C	0,0	187	C	0,0	103	C	0,0	134	C	0,0	
176	Q	0,0	150	Q	0,0	188	Q	0,0	104	Q	0,0	135	Q	0,0	
177	V	0,0	151	V	0,0	189	I	0,0	105	A	0,0	136	A	0,0	LBD H3
178	V	0,0	152	V	0,0	190	G	0,0	106	T	0,0	137	T	0,0	
179	N	0,0	153	N	0,0	191	N	0,0	107	H	0,1	138	N	0,0	
180	K	0,0	154	K	0,0	192	K	0,0	108	K	0,0	139	K	0,0	
181	Q	0,0	155	Q	0,0	193	Q	0,0	109	Q	0,0	140	Q	0,0	
182	L	0,0	156	L	0,0	194	I	0,0	110	L	0,0	141	L	0,0	
183	F	0,1	157	F	0,0	195	A	0,0	111	F	0,0	142	F	0,0	
184	Q	0,0	158	Q	0,0	196	A	0,0	112	Q	0,0	143	Q	0,0	
185	M	0,0	159	M	0,0	197	L	0,0	113	L	0,0	144	L	0,0	
186	V	0,0	160	V	0,0	198	V	0,1	114	V	0,0	145	V	0,0	
187	E	0,0	161	E	0,0	199	V	0,0	115	Q	0,0	146	E	0,0	
188	Y	0,0	162	Y	0,0	200	W	0,0	116	W	0,0	147	W	0,0	
189	A	0,0	163	A	0,1	201	A	0,0	117	A	0,0	148	A	0,0	
190	R	0,0	164	R	0,0	202	R	0,0	118	K	0,0	149	K	0,0	
191	M	0,1	165	M	0,1	203	D	0,0	119	L	0,1	150	H	0,0	
192	M	0,0	166	M	0,1	204	I	0,0	120	V	0,1	151	I	0,0	
193	P	0,0	167	P	0,0	205	P	0,0	121	P	0,0	152	P	0,0	
194	H	0,0	168	H	0,0	206	H	0,0	122	H	0,0	153	H	0,0	
195	F	0,0	169	F	0,0	207	F	0,0	123	F	0,0	154	F	0,0	
196	A	0,0	170	A	0,1	208	S	0,3	124	T	0,0	155	T	0,0	
197	Q	0,0	171	Q	0,1	209	Q	0,0	125	S	0,0	156	T	0,2	
198	V	0,1	172	V	0,0	210	L	0,0	126	L	0,0	157	L	0,0	
199	P	0,0	173	P	0,1	211	E	0,0	127	P	0,0	158	P	0,0	
200	L	0,0	174	L	0,1	212	M	0,3	128	L	0,1	159	L	0,0	
201	D	0,0	175	D	0,1	213	E	0,2	129	T	0,1	160	S	1,0	
202	D	0,0	176	D	0,0	214	D	0,0	130	D	0,0	161	D	0,0	
203	Q	0,0	177	Q	0,0	215	Q	0,0	131	Q	0,0	162	Q	0,0	

204	V	0,0	178	V	0,1	216	I	0,2	132	V	0,0	163	V	0,0	
205	I	0,0	179	I	0,1	217	L	0,3	133	Q	0,1	164	L	0,0	
206	L	0,0	180	L	0,0	218	L	0,0	134	L	0,0	165	L	0,0	LBD H4-H5
207	L	0,0	181	L	0,0	219	I	0,0	135	L	0,0	166	L	0,0	
208	K	0,0	182	K	0,0	220	K	0,0	136	R	0,0	167	R	0,0	
209	A	0,0	183	A	0,1	221	G	0,3	137	A	0,0	168	A	0,0	
210	A	0,1	184	A	0,1	222	S	0,1	138	G	0,0	169	G	0,0	
211	W	0,0	185	W	0,0	223	W	0,0	139	W	0,0	170	W	0,0	
212	I	0,1	186	I	0,0	224	N	0,0	140	N	0,0	171	N	0,0	
213	E	0,0	187	E	0,0	225	E	0,0	141	E	0,0	172	E	0,0	
214	L	0,0	188	L	0,0	226	L	0,0	142	L	0,0	173	L	0,0	
215	L	0,0	189	L	0,0	227	L	0,0	143	L	0,0	174	L	0,0	
216	I	0,0	190	I	0,0	228	L	0,0	144	I	0,0	175	I	0,0	
217	A	0,0	191	A	0,0	229	F	0,0	145	A	0,0	176	A	0,0	
218	N	0,0	192	N	0,1	230	A	0,0	146	A	0,0	177	A	0,0	
219	V	0,0	193	V	0,0	231	I	0,0	147	F	0,0	178	F	0,0	
220	A	0,0	194	A	0,0	232	A	0,0	148	S	0,0	179	S	0,0	
221	W	0,0	195	W	0,0	233	W	0,0	149	H	0,0	180	H	0,0	
222	C	0,1	196	C	0,1	234	R	0,0	150	R	0,0	181	R	0,0	
223	S	0,0	197	S	0,0	235	S	0,0	151	S	0,0	182	S	0,0	
224	I	0,0	198	I	0,0	236	M	0,0	152	M	0,0	183	V	0,0	
												184	E	0,0	
												185	V	0,0	
												186	K	0,0	
									153	Q	0,0				
									154	A	0,0				
									155	Q	0,0				
225	V	0,1	199	V	0,1	237	E	0,0							
226	S	0,0	200	S	0,0	238	F	0,3							
227	L	0,1	201	L	0,1	239	L	0,0							
228	*D*	*0,0*	*202*	*D*	*0,0*	240	T	0,3							
229	*D*	*0,1*	*203*	*D*	*0,1*	241	E	0,1							
						242	*E*	*0,0*							
						243	*R*	*0,0*							
						244	*D*	*0,2*							
						245	*G*	*0,2*							
						246	*V*	*0,2*							
230	*G*	*0,0*													
231	*L*	*0,2*													
232	*G*	*0,1*													
233	*H*	*0,0*													residues in talics and boxed:
234	*D*	*0,0*				*247*	*D*	*0,0*							not stabilised in the crystals
235	*G*	*0,1*													
236	*S*	*0,1*				*248*	*T*	*0,2*							
237	*F*	*0,0*													
238	*E*	*0,1*													
239	*R*	*0,0*													
240	*R*	*0,0*				249	R	0,0							
						250	T	0,2							
						251	T	0,0							
						252	S	0,2							
						253	P	0,1							
241	*S*	*0,0*	*204*	*S*	*0,1*										
242	*P*	*0,0*	*205*	*P*	*0,1*										
243	*G*	*0,1*	*206*	*G*	*0,1*										
244	*L*	*0,1*	*207*	*L*	*0,1*										
245	Q	0,0	208	Q	0,0										
246	P	0,0	209	P	0,0	254	P	0,0							
247	Q	0,0	210	Q	0,0	255	Q	0,0							
248	Q	0,0	211	Q	0,0	256	L	0,0							
249	L	0,0	212	L	0,0	257	M	0,1							ß-s1
250	F	0,0	213	F	0,0	258	C	0,0							
									156	D	0,0	187	D	0,0	
									157	A	0,0	188	G	0,0	
									158	I	0,0	189	I	0,0	
									159	V	0,0	190	V	0,0	
251	L	0,0	214	L	0,0	259	L	0,0	160	L	0,0	191	L	0,0	
252	N	0,1	215	N	0,1	260	M	0,0	161	A	0,0	192	A	0,0	
253	Q	0,0	216	Q	0,0	261	P	0,0	162	T	0,0	193	T	0,0	
254	S	0,1	217	S	0,0	262	G	0,0	163	G	0,0	194	G	0,0	

255	F	0,0	218	F	0,0	263	M	0,0	164	L	0,0	195	L	0,0	
256	S	0,0	219	S	0,0	264	T	0,0	165	T	0,0	196	T	0,0	
257	Y	0,0	220	Y	0,1	265	L	0,0	166	V	0,0	197	V	0,0	ß-s2
258	H	0,0	221	H	0,0	266	H	0,0	167	N	0,0	198	H	0,0	
259	R	0,0	222	R	0,0	267	R	0,0	168	K	0,0	199	R	0,0	
260	N	0,0	223	N	0,0	268	N	0,0	169	S	0,1	200	N	0,0	
261	S	0,0	224	S	0,0	269	S	0,0	170	T	0,0	201	S	0,0	
262	A	0,0	225	A	0,0	270	A	0,0	171	A	0,0	202	A	0,0	
263	I	0,1	226	I	0,1	271	L	0,1	172	H	0,1	203	H	0,0	
264	K	0,0	227	K	0,0	272	Q	0,0	173	A	0,0	204	Q	0,0	
265	A	0,0	228	A	0,0	273	A	0,0	174	V	0,0	205	A	0,0	
266	G	0,0	229	G	0,1	274	G	0,0	175	G	0,0	206	G	0,0	
267	V	0,0	230	V	0,0	275	V	0,0	176	V	0,0	207	V	0,0	
268	S	0,1	231	S	0,1	276	G	0,0	177	G	0,0	208	G	0,0	
269	A	0,2	232	A	0,1	277	Q	0,2	178	N	0,0	209	A	0,1	
270	I	0,0	233	I	0,0	278	I	0,0	179	I	0,0	210	I	0,0	
271	F	0,0	234	F	0,0	279	F	0,0	180	Y	0,0	211	F	0,0	
272	D	0,0	235	D	0,0	280	D	0,0	181	D	0,0	212	D	0,0	
273	R	0,0	236	R	0,0	281	R	0,0	182	R	0,0	213	R	0,0	LBD H6-H7
274	I	0,0	237	I	0,0	282	V	0,0	183	V	0,0	214	V	0,0	
275	L	0,0	238	L	0,0	283	L	0,0	184	L	0,0	215	L	0,0	
276	S	0,0	239	S	0,0	284	S	0,0	185	S	0,0	216	T	0,0	
277	E	0,0	240	E	0,0	285	E	0,0	186	E	0,0	217	E	0,0	
278	L	0,0	241	L	0,0	286	L	0,0	187	L	0,0	218	L	0,0	
279	S	0,0	242	S	0,0	287	S	0,3	188	V	0,0	219	V	0,0	
280	V	0,0	243	V	0,0	288	L	0,0	189	N	0,0	220	A	0,0	
281	K	0,0	244	K	0,0	289	K	0,0	190	K	0,0	221	K	0,0	
282	M	0,0	245	M	0,0	290	M	0,0	191	M	0,0	222	M	0,0	
283	K	0,0	246	K	0,0	291	R	0,0	192	K	0,0	223	R	0,0	
284	R	0,1	247	R	0,0	292	T	0,3	193	E	0,0	224	E	0,0	
285	L	0,0	248	L	0,0	293	L	0,0	194	M	0,0	225	M	0,0	
286	N	0,0	249	N	0,0	294	R	0,1	195	K	0,0	226	K	0,0	
287	L	0,0	250	L	0,1	295	V	0,2	196	M	0,0	227	M	0,0	
288	D	0,0	251	D	0,0	296	D	0,0	197	D	0,0	228	D	0,0	
289	R	0,0	252	R	0,1	297	Q	0,0	198	K	0,0	229	K	0,0	
290	R	0,1	253	R	0,1	298	A	0,0	199	T	0,0	230	T	0,0	
291	E	0,0	254	E	0,0	299	E	0,0	200	E	0,0	231	E	0,0	
292	L	0,0	255	L	0,0	300	Y	0,1	201	L	0,0	232	L	0,0	
293	S	0,2	256	S	0,1	301	V	0,0	202	G	0,0	233	G	0,0	LBD H8
294	C	0,0	257	C	0,1	302	A	0,0	203	C	0,0	234	C	0,0	
295	L	0,0	258	L	0,0	303	L	0,0	204	L	0,0	235	L	0,0	
296	K	0,0	259	K	0,0	304	K	0,0	205	R	0,0	236	R	0,0	
297	A	0,0	260	A	0,0	305	A	0,0	206	A	0,0	237	S	0,0	
298	I	0,0	261	I	0,0	306	I	0,1	207	I	0,0	238	V	0,0	
299	I	0,0	262	I	0,0	307	I	0,1	208	I	0,0	239	I	0,0	
300	L	0,0	263	L	0,0	308	L	0,0	209	L	0,0	240	L	0,0	
301	Y	0,0	264	Y	0,0	309	L	0,0	210	Y	0,0	241	F	0,0	
302	N	0,0	265	N	0,0	310	N	0,0	211	N	0,0	242	N	0,0	
303	P	0,1	266	P	0,0	311	P	0,0	212	P	0,0	243	P	0,0	
304	D	0,0	267	D	0,0	312	D	0,0	213	D	0,0	244	D	0,0	
305	I	0,1	268	I	0,0	313	V	0,1	214	V	0,0	245	V	0,0	
306	R	0,0	269	R	0,0	314	K	0,0	215	R	0,0	246	R	0,0	
307	G	0,0	270	G	0,0	315	G	0,0	216	G	0,0	247	G	0,0	
308	I	0,0	271	I	0,0	316	L	0,0	217	I	0,0	248	L	0,0	
309	K	0,0	272	K	0,0	317	K	0,2	218	K	0,0	249	K	0,0	
310	S	0,1	273	S	0,1	318	N	0,1	219	S	0,0	250	S	0,0	
311	R	0,0	274	R	0,0	319	R	0,1	220	V	0,0	251	S	1,0	
312	A	0,1	275	A	0,1	320	Q	0,1	221	Q	0,0	252	Q	0,2	
313	E	0,1	276	E	0,1	321	E	0,0	222	E	0,0	253	E	0,1	
314	I	0,0	277	I	0,1	322	V	0,0	223	V	0,0	254	V	0,0	
315	E	0,0	278	E	0,0	323	E	0,2	224	E	0,0	255	E	0,0	
316	M	0,1	279	M	0,2	324	V	0,0	225	M	0,0	256	L	1,0	
317	C	0,0	280	C	0,1	325	L	0,0	226	L	0,0	257	L	0,0	
318	R	0,0	281	R	0,0	326	R	0,0	227	R	0,0	258	R	0,0	
319	E	0,0	282	E	0,0	327	E	0,0	228	E	0,0	259	E	0,0	
320	K	0,0	283	K	0,0	328	K	0,0	229	K	0,0	260	K	0,0	
321	V	0,0	284	V	0,0	329	M	0,0	230	I	0,0	261	V	0,0	LBD H9
322	Y	0,0	285	Y	0,0	330	F	0,1	231	Y	0,0	262	Y	0,0	

323	A	0,0	286	A	0,0	331	L	0,2	232	G	0,0	263	A	0,0	
324	C	0,0	287	C	0,0	332	C	0,0	233	V	0,0	264	A	0,0	
325	L	0,0	288	L	0,0	333	L	0,0	234	L	0,0	265	L	0,0	
326	D	0,0	289	D	0,0	334	D	0,0	235	E	0,0	266	E	0,0	
327	E	0,0	290	E	0,0	335	E	0,1	236	E	0,0	267	E	0,0	
328	H	0,0	291	H	0,0	336	Y	0,0	237	Y	0,0	268	Y	0,0	
329	C	0,0	292	C	0,0	337	C	0,2	238	T	0,0	269	T	0,0	
330	R	0,0	293	R	0,0	338	R	0,0	239	R	0,0	270	R	0,0	
331	L	0,2	294	L	0,9	339	R	0,0	240	T	0,0	271	T	0,0	
332	E	0,0	295	E	0,0	340	S	0,2	241	T	0,0	272	T	0,0	
333	H	0,0	296	H	0,0	341	R	0,0	242	H	0,0	273	Y	0,0	
334	P	0,0	297	P	0,0	342	S	0,3	243	P	0,0	274	P	0,0	
335	G	0,0	298	G	0,1	343	S	0,3	244	N	0,0	275	D	0,0	
336	D	0,0	299	D	0,0	344	E	0,0	245	E	0,0	276	E	0,0	
337	D	0,0	300	D	0,0	345	E	0,0	246	P	0,0	277	P	0,0	
338	G	0,0	301	G	0,0	346	G	0,0	247	G	0,0	278	G	0,0	
339	R	0,0	302	R	0,0	347	R	0,0	248	R	0,0	279	R	0,0	
340	F	0,0	303	F	0,0	348	F	0,0	249	F	0,0				
341	A	0,0	304	A	0,0	349	A	0,0	250	A	0,0				
342	Q	0,0	305	Q	0,0	350	A	0,2	251	K	0,0				
343	L	0,0	306	L	0,0	351	L	0,0	252	L	0,0				
344	L	0,0	307	L	0,0	352	L	0,0	253	L	0,0				
345	L	0,0	308	L	0,0	353	L	0,0	254	L	0,0				
346	R	0,0	309	R	0,0	354	R	0,0	255	R	0,0				
347	L	0,0	310	L	0,0	355	L	0,0	256	L	0,0				
348	P	0,0	311	P	0,0	356	P	0,0	257	P	0,0				
349	A	0,0	312	A	0,0	357	A	0,0	258	A	0,0				
350	L	0,0	313	L	0,0	358	L	0,0	259	L	0,0				
351	R	0,0	314	R	0,0	359	R	0,0	260	R	0,0				LBD H10-H11
352	S	0,0	315	S	0,0	360	S	0,0	261	S	0,0				
353	I	0,0	316	I	0,0	361	I	0,0	262	I	0,0				
354	S	0,0	317	S	0,0	362	S	0,0	263	G	0,0				
355	L	0,0	318	L	0,0	363	L	0,0	264	L	0,0				
356	K	0,0	319	K	0,0	364	K	0,0	265	K	0,0				
357	C	0,0	320	C	0,0	365	S	0,1	266	C	0,0				
358	Q	0,1	321	Q	0,1	366	F	0,0	267	L	0,0				
359	D	0,0	322	D	0,0	367	E	0,0	268	E	0,0				
360	H	0,0	323	H	0,0	368	H	0,0	269	H	0,0				
361	L	0,1	324	L	0,0	369	L	0,0	270	L	0,0				
362	F	0,0	325	F	0,0	370	F	0,1	271	F	0,0				
363	L	0,1	326	L	0,0	371	F	0,1	272	F	0,0				
364	F	0,0	327	F	0,0	372	F	0,0	273	F	0,0				
365	R	0,1	328	R	0,0	373	H	0,0	274	K	0,0				
366	I	0,0	329	I	0,0	374	L	0,0	275	L	0,0				
367	T	0,1	330	T	0,1	375	V	0,0	276	I	0,0				
368	S	0,0	331	S	0,1	376	A	0,0	277	G	0,0				
369	D	0,0	332	D	0,0	377	D	0,1	278	D	0,0				
370	R	0,0	333	R	0,0	378	T	0,3	279	V	0,0				
						379	S	0,2							
						380	I	0,1							
						381	A	0,3							
						382	G	0,2							
						383	Y	0,0							
						384	I	0,0							
						385	R	0,1							
						386	D	0,1							
						387	A	0,2							
						388	L	0,0							
						389	R	0,1							
						390	N	0,2							
						391	H	0,0							
						392	A	0,2							
						393	P	0,0							
371	P	0,0	334	P	0,1	394	P	0,1	280	P	0,0				
372	L	0,0	335	L	0,0	395	I	0,1	281	I	0,0				
373	E	0,1	336	E	0,0	396	D	0,0	282	D	0,0				
374	E	0,1	337	E	0,1	397	T	0,3	283	T	0,0				
375	L	0,0	338	L	0,1	398	N	0,3							

376	F	0,0	339	F	0,0				LBD H12
377	L	0,1	340	L	0,1				
			341	E	0,0				
			342	Q	0,1				
			343	L	0,0				
			344	E	0,0				
			345	A	0,1				
			346	P	0,1				

Table S3. dN/dS of usp in Drosophilidae, Diptera, Lepidoptera, Tenebrionidae and Blattidae.
