# Supplementary material for: Molecular adaptation and resilience of the insect’s nuclear receptor USP
Source: BMC Evol Biol. 2012 Oct 5;12:199. doi: 10.1186/1471-2148-12-199 (PMC3520820; doi:10.1186/1471-2148-12-199)
Supplement: Additional file 3 — Table S4. Standard errors [SE] associated to the parameters of the models fitted in the likelihood analysis reported in Table 1. [file 1471-2148-12-199-S3.pdf]

Model		Drosophilidae	Diptera	Lepidoptera	Tenebrionidae	Blattaria
M1a						
	LnL *(np)*	-5649.5 *(31)*	-6946.6 *(25)*	-4376.2 *(12)*	-2113.5 *(10)*	-1945.6 *(13)*
	ω_0[SE]	0.020 [0.002]	0.026 [0.003]	0.028 [0.003]	0.009 [0.003]	0.004 [0.002]
	p_0[SE]	0.983 [0.007]	0.976 [0.009]	0.952 [0.013]	0.992 [0.006]	0.985 [0.008]
	LnL *(np)*	-5649.5 (*33*)	-6946.6 *(27)*	-4376.2 *(14)*	-2113.5 (*12*)	-1945.6 (*15*)
M2a	ω_0[SE]	0.020 [0.002]	0.026 [0.003]	0.028 [0.003]	0.009 [0.003]	0.004 [0.002]
	ω_2[SE]	1.000 [0.000]	1.000 [0.000]	1.000 [na]	1.000 [0.000]	1.237 [na]
	p_0[SE]	0.983 [0.007]	0.976 [0.009]	0.952 [0.013]	0.992 [0.006]	0.985 [0.008]
	p_1[SE]	0.011 [0.007]	0.010 [0.007]	0.029 [0.013]	0.005 [0.004]	0.006 [na]
M7	LnL *(np)*	-5610.8 *(31)*	-6859.5 *(25)*	-4339.9 *(12)*	-2112.9 *(10)*	-1950.0 *(13)*
	p [SE]	0.159 [0.027]	0.404 [0.055]	0.150 [0.026]	0.059 [0.062]	0.011 [0.055]
	q [SE]	5.048 [0.900]	10.774 [2.037]	2.578 [0.560]	3.129 [4.238]	0.308 [2.024]
M8	LnL *(np)*	-5610.8 *(33)*	-6857.1 *(27)*	-4338.8 *(14)*	-2112.9 *(12)*	-1946 *(15)*
	p [SE]	0.159 [0.028]	0.432 [0.0621]	0.156 [0.028]	0.059 [0.062]	0.023 [0.212]
	q [SE]	5.049 [0.955]	12.880 [2.824]	2.85 [0.630]	3.133 [4.282]	2.089 [49.209]
	ω_s[SE]	1.000 [na]	1.000 [1.196]	4.457 [8.024]	1.000 [na]	1.000 [0.607]
	p_0[SE]	1.000 [na]	0.994 [0.007]	0.997 [0.003]	1.000 [0.000]	0.985 [0.009]

Table S4: Standard errors [SE] associated to the parameters of the models fitted in the likelihood analysis reported in Table 1.
