# Supplementary figures and images for: Molecular adaptation and resilience of the insect’s nuclear receptor USP
Source: BMC Evol Biol. 2012 Oct 5;12:199. doi: 10.1186/1471-2148-12-199 (PMC3520820; doi:10.1186/1471-2148-12-199)

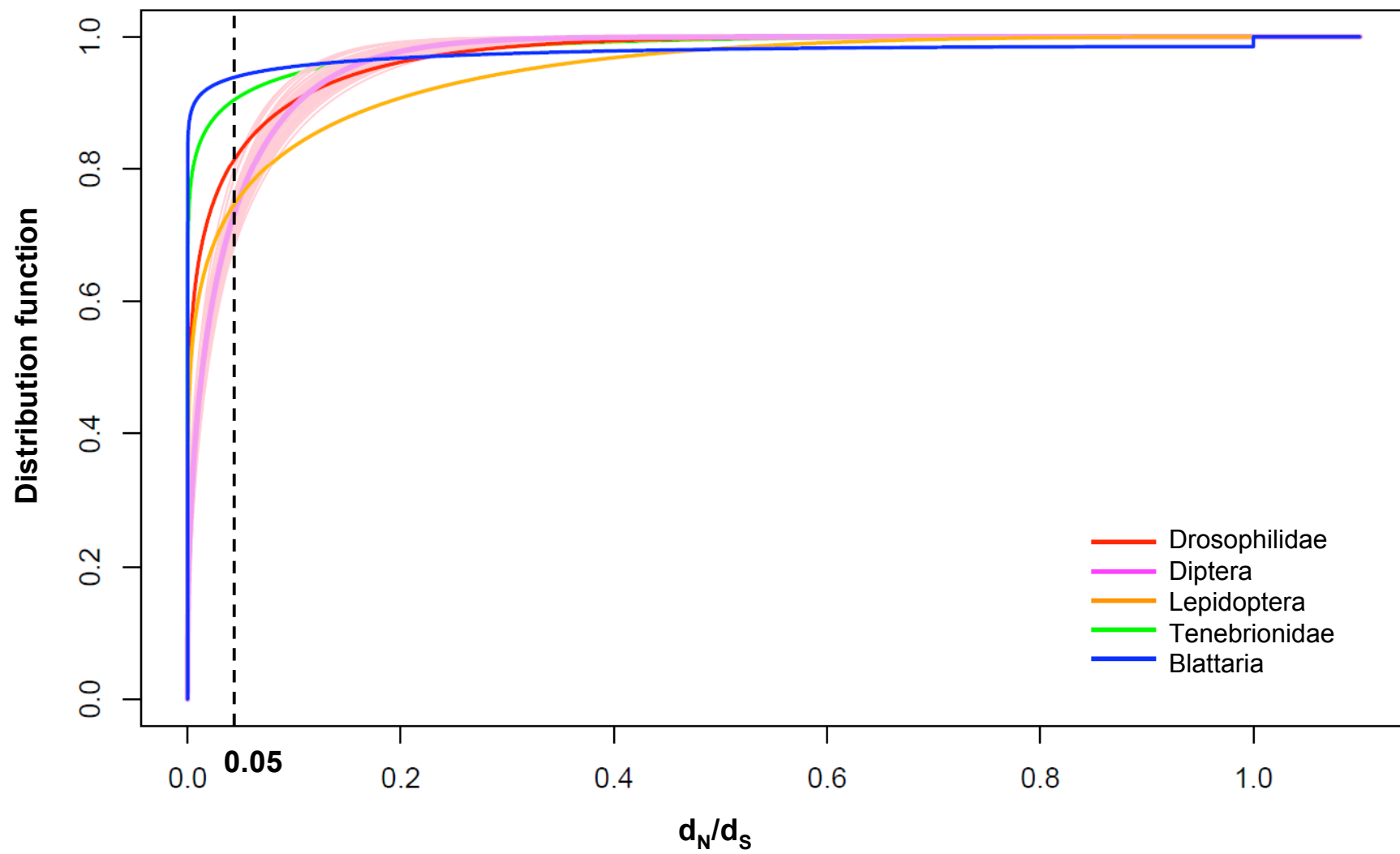

Supplement: Additional file 4 — Figure S1. Robustness of dN/dS estimates to saturation in Diptera usp dataset. The accuracy of the estimate of the β distribution function was tested by re-performing the Codeml analysis (under model M7) on 50 simulated alignments, which were generated with Evolver in the PAML package using the global parameters established for the real usp Diptera dataset (number of sequences, sequence length, topology and branch lengths of the tree, β distribution function for dN/dS, Ts/Tv ratio, codon usage). Bold lines are the same as in Figure 2: Drosophilidae: red; Diptera: purple; Lepidoptera: orange; Tenebrionidae: green; Blattaria: blue. A thin pink line was added for each ones of the 50 re-analysis of the simulated alignments. [file 1471-2148-12-199-S4.pdf]

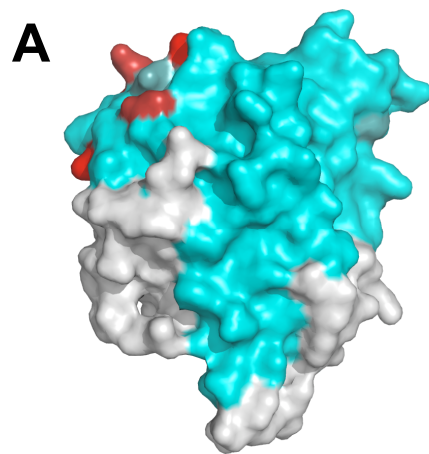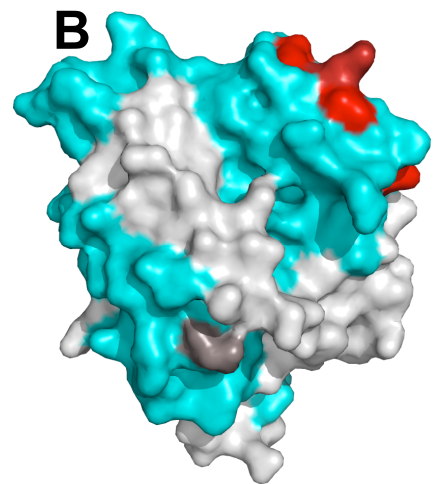

180°  
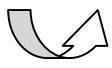  
**Blattaria**

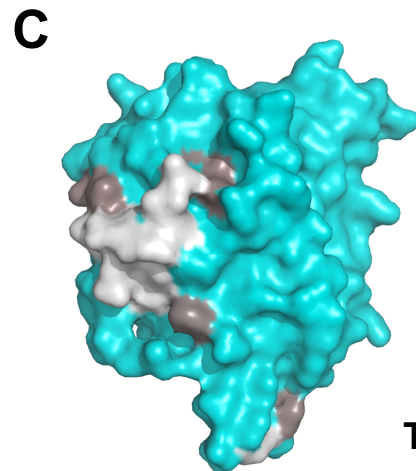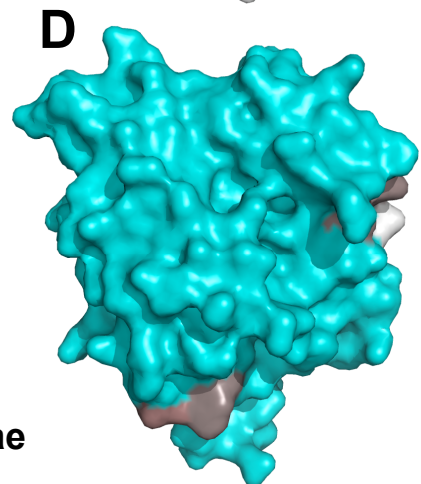

180°  
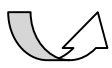  
**Tenebrionidae**

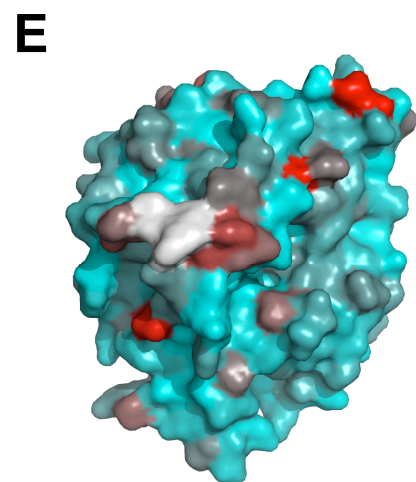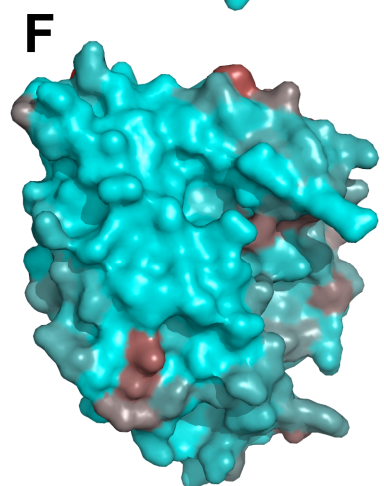

180°  
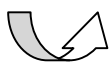  
**Diptera**

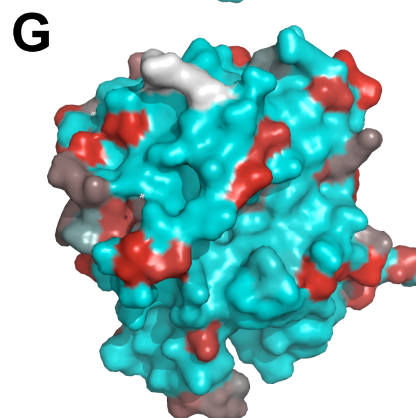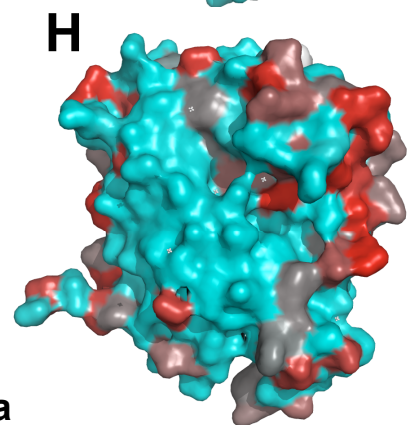

180°  
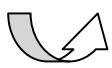  
**Lepidoptera**

Supplement: Additional file 6 — Figure S2. Evolutionary rates on the surface of USP structures in four extant groups of insects. Site-specific dN/dS were projected onto the crystal structure of the USP LBD domain of Tribolium (2NXX, ECR-USP) for Blattaria (A) and Tenebrionidae (B), of Drosophila (1HG4, USP) for Diptera (C) and of Heliothis (1R1K, ECR-USP) for Lepidoptera (D). The values are distributed along a colour scale from blue (low dN/dS) to red (high dN/dS). Sequences not available for the estimation of evolutionary rates are in white. Views on the left show the side of the LBD that is near ECR, while views on the right show the same structure after a rotation of 180°. [file 1471-2148-12-199-S6.pdf]

## Slide 1
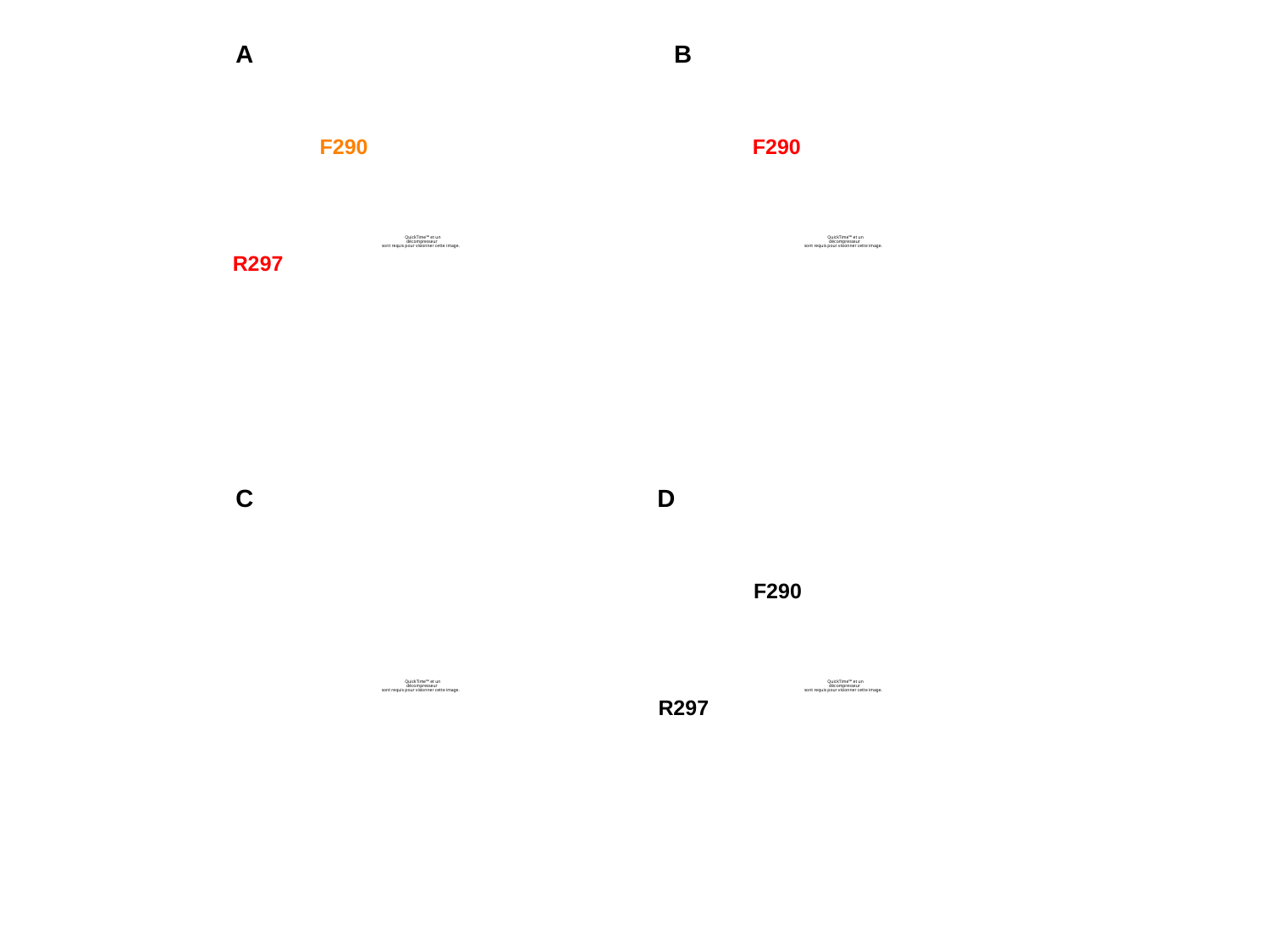

A
B
F290
F290
R297
C
D
F290
R297

Supplement: Additional file 8 — Figure S3. Selection in the putative coactivation surface during the radiation of Mecopterida. Posterior probabilities for each site to belong to the site-class under positive selection (along branch A or branch B) or relaxed evolution (along branch C) were projected onto the crystal structure of the USP LBD domain of Drosophila (1HG4). Probabilities are distributed along a colour scale from yellow (p=0) to red (p=1). Sequences not available for the estimation of evolutionary rates are in white. (A) Branch A, stem lineage of Mecopterida. (B) Branch B, subdivision between Amphiesmenoptera (Lepidoptera, Trichoptera) and Antliophora (Diptera, Mecoptera, Siphonaptera). (C) Branch C, subdivision of Diptera into the suborders Brachycera and Nematocera. (D) The coactivation surface, defined by homology with RXR, is shown in pink. The helix H12 is showed in green. [file 1471-2148-12-199-S8.ppt]
