# Supplementary material for: Molecular adaptation and resilience of the insect’s nuclear receptor USP
Source: BMC Evol Biol. 2012 Oct 5;12:199. doi: 10.1186/1471-2148-12-199 (PMC3520820; doi:10.1186/1471-2148-12-199)
Supplement: Additional file 5 — Table S5. Comparison between usp and cox1 substitution rates. [file 1471-2148-12-199-S5.doc]

|  |  | *usp* | *cox1* | *usp/cox1* |
| --- | --- | --- | --- | --- |
| Drosophilidae | d_N_ | 0,176 | 0,051 | 3,4 |
|  | d_S_ | 6,538 | 13,231 | 0,5 |
|  | d_N_/d_S_ | 0,027 | 0,004 | 7,0 |
| Tenebrionidae | d_N_ | 0,026 | 0,066 | 0,4 |
|  | d_S_ | 2,020 | 7,753 | 0,3 |
|  | d_N_/d_S_ | 0,013 | 0,008 | 1,5 |
| Blattaria | d_N_ | 0,044 | 0,056 | 0,8 |
|  | d_S_ | 3,031 | 13,721 | 0,2 |
|  | d_N_/d_S_ | 0,014 | 0,004 | 3,5 |

**Table S5.** Comparison between *usp* and *cox1* substitution rates.
