# Supplementary material for: Molecular adaptation and resilience of the insect’s nuclear receptor USP
Source: BMC Evol Biol. 2012 Oct 5;12:199. doi: 10.1186/1471-2148-12-199 (PMC3520820; doi:10.1186/1471-2148-12-199)
Supplement: Additional file 7 — Table S6. Posterior probabilities for each site to belong to the site-class under positive selection (along branch A or branch B) or relaxation (along branch C). [file 1471-2148-12-199-S7.pdf]

Branch A			Branch B			Branch C			
1	C	0,0	1	C	0,0	31	C	0,0	
2	E	0,0	2	E	0,0	32	E	0,0	DBD Zinc-finger 1(partial)
3	G	0,0	3	G	0,0	33	G	0,0	
4	C	0,0	4	C	0,0	34	C	0,0	
5	K	0,0	5	K	0,0	35	K	0,0	
6	G	0,0	6	G	0,0	36	G	0,0	
7	F	0,0	7	F	0,0	37	F	0,0	
8	F	0,0	8	F	0,0	38	F	0,0	
9	K	0,0	9	K	0,0	39	K	0,0	
10	R	0,0	10	R	0,0	40	R	0,0	
11	T	0,0	11	T	0,0	41	T	0,0	
12	V	0,0	12	V	0,0	42	V	0,0	
13	R	0,0	13	R	0,0	43	R	0,0	
14	K	0,0	14	K	0,0	44	K	0,0	
15	D	0,0	15	D	0,0	45	D	0,0	
16	L	0,1	16	L	0,0	46	L	0,0	
17	T	0,1	17	T	0,1	47	T	0,5	
18	Y	0,0	18	Y	0,0	48	Y	0,0	
19	A	0,0	19	A	0,0	49	A	0,0	
20	C	0,0	20	C	0,0	50	C	0,0	
21	R	0,0	21	R	0,0	51	R	0,0	
22	E	0,0	22	E	0,0	52	E	0,0	
23	N	0,0	23	N	0,2	53	N	0,0	
24	R	0,4	24	R	0,0	54	R	0,1	
25	N	0,0	25	N	0,0	55	N	0,0	
26	C	0,0	26	C	0,0	56	C	0,0	
27	I	0,1	27	I	0,0	57	I	0,1	
28	I	0,0	28	I	0,0	58	I	0,0	
29	D	0,0	29	D	0,0	59	D	0,0	DBD Zinc-finger 2
30	K	0,0	30	K	0,0	60	K	0,0	
31	R	0,0	31	R	0,0	61	R	0,0	
32	Q	0,0	32	Q	0,0	62	Q	0,0	
33	R	0,0	33	R	0,0	63	R	0,0	
34	N	0,0	34	N	0,0	64	N	0,0	
35	R	0,1	35	R	0,0	65	R	0,0	
36	C	0,0	36	C	0,0	66	C	0,0	
37	Q	0,0	37	Q	0,0	67	Q	0,0	
38	Y	0,0	38	Y	0,0	68	Y	0,0	
39	C	0,0	39	C	0,0	69	C	0,0	
40	R	0,1	40	R	0,0	70	R	0,0	
41	Y	0,0	41	Y	0,0	71	Y	0,0	
42	Q	0,0	42	Q	0,0	72	Q	0,0	
43	K	0,0	43	K	0,0	73	K	0,0	
44	C	0,0	44	C	0,0	74	C	0,0	
45	L	0,1	45	L	0,0	75	L	0,0	

46 T	0,0	46 T	0,0	76 T	0,0	
47 C	1,0	47 C	0,0	77 C	0,0	
48 G	0,0	48 G	0,0	78 G	0,0	
49 M	0,0	49 M	0,0	79 M	0,0	
50 K	0,0	50 K	0,0	80 K	0,0	
51 R	0,1	51 R	0,0	81 R	0,0	
52 E	0,0	52 E	0,0	82 E	0,0	
53 A	0,0	53 A	0,0	83 A	0,0	
54 V	0,0	54 V	0,0	84 V	0,0	
55 Q	0,0	55 Q	0,0	85 Q	0,0	DBD T-box
56 E	0,0	56 E	0,0	86 E	0,0	
57 E	0,0	57 E	0,0	87 E	0,0	
58 R	0,0	58 R	0,0	88 R	0,0	
59 Q	0,0	59 Q	0,0	89 Q	0,0	
60 R	0,1	60 R	0,0	90 R	0,0	
61 G	1,0	61 G	0,6	91 G	0,0	
62 A	1,0	62 A	0,0	92 A	0,1	
63 R	0,6	63 R	0,0	93 R	0,7	
64 D	1,0	64 D	0,0	94 D	0,0	
65 D	0,0	65 D	0,0	95 D	0,0	
66 F	0,1	66 F	1,0	96 F	0,4	
67 M	1,0	67 M	0,4	97 M	0,3	D domain
68 T	1,0	68 T	0,0	98 T	0,2	
69 N	0,1	69 N	0,6	99 N	0,4	
70 S	0,0	70 S	0,0	100 S	0,0	
71 V	1,0	71 V	0,0	101 V	0,0	
72 S	0,9	72 S	0,0	102 S	0,1	
				103 R	0,0	
73 D	0,0	73 D	0,3	104 D	0,0	
74 F	1,0	74 F	0,0	105 F	0,3	
75 S	1,0	75 S	0,0	106 S	0,0	
76 I	0,0	76 I	0,0	107 I	0,0	
77 E	0,0	77 E	0,0	108 E	0,0	
78 R	0,1	78 R	0,0	109 R	0,0	
79 I	0,0	79 I	0,7	110 I	0,0	LBD H1
80 I	0,0	80 I	0,0	111 I	0,0	
81 E	0,0	81 E	0,0	112 E	0,0	
82 A	0,1	82 A	1,0	113 A	0,0	
83 E	0,0	83 E	0,0	114 E	0,0	
				115 Q	0,0	
				116 R	0,3	
				117 A	0,9	
				118 E	0,0	
				119 T	0,1	
				120 Q	0,0	
				121 C	0,0	

				122 G	0,0	
				123 D	0,0	
				124 R	0,0	
				125 A	0,0	
		84 L	0,5	126 L	0,6	
		85 T	0,0	127 T	0,0	
		86 F	0,0	128 F	0,3	
		87 L	0,0	129 L	0,0	
		88 R	0,0	130 R	0,0	
		89 V	0,0	131 V	0,0	
		90 G	0,0	132 G	0,0	
		91 P	0,0	133 P	0,3	
		92 Y	0,1	134 Y	0,0	
		93 S	0,0	135 S	0,0	
		94 T	0,1	136 T	0,0	
		95 V	0,0	137 V	0,3	
		96 Q	0,0	138 Q	0,5	
		97 P	0,0	139 P	0,0	
		98 D	0,0	140 D	0,2	
		99 Y	0,0	141 Y	0,0	
		100 K	0,8	142 K	0,0	
		101 G	0,0	143 G	0,0	
		102 A	0,0	144 A	0,0	
84 V	0,0	103 V	0,0	145 V	0,0	
85 S	0,6	104 S	0,0	146 S	0,0	
86 A	0,2	105 A	0,8	147 A	0,0	
87 L	1,0	106 L	0,0	148 L	0,0	
88 C	0,0	107 C	0,0	149 C	0,0	
89 Q	0,0	108 Q	0,0	150 Q	0,0	LBD H3
90 V	1,0	109 V	0,5	151 V	0,0	
91 V	1,0	110 V	0,5	152 V	0,0	
92 N	0,0	111 N	0,0	153 N	0,0	
93 K	0,0	112 K	0,0	154 K	0,0	
94 Q	0,0	113 Q	0,0	155 Q	0,0	
95 L	0,1	114 L	0,0	156 L	0,3	
96 F	0,6	115 F	0,9	157 F	0,0	
97 Q	0,1	116 Q	1,0	158 Q	0,0	
98 M	0,1	117 M	0,0	159 M	0,4	
99 V	0,0	118 V	0,0	160 V	0,3	
100 E	0,0	119 E	0,3	161 E	0,1	
101 Y	0,0	120 Y	1,0	162 Y	0,3	
102 A	0,0	121 A	0,0	163 A	0,0	
103 R	1,0	122 R	0,0	164 R	0,0	
104 M	1,0	123 M	0,0	165 M	0,0	
105 M	0,1	124 M	0,1	166 M	0,6	
106 P	0,0	125 P	0,0	167 P	0,0	

107	H	0,0	126	H	0,0	168	H	0,0	
108	F	0,0	127	F	0,0	169	F	0,0	
109	A	0,5	128	A	0,0	170	A	0,0	
110	Q	1,0	129	Q	0,8	171	Q	0,4	
111	V	0,1	130	V	0,0	172	V	0,0	
112	P	0,8	131	P	0,0	173	P	0,0	
113	L	0,1	132	L	0,0	174	L	0,0	
114	D	0,0	133	D	0,1	175	D	0,0	
115	D	0,0	134	D	0,0	176	D	0,0	
116	Q	0,0	135	Q	0,0	177	Q	0,0	
117	V	0,0	136	V	0,0	178	V	0,0	
118	I	0,1	137	I	0,4	179	I	0,1	
119	L	0,1	138	L	0,0	180	L	0,0	
120	L	0,1	139	L	0,0	181	L	0,0	
121	K	0,1	140	K	0,0	182	K	0,0	LBD H4-H5
122	A	0,0	141	A	0,0	183	A	0,9	
123	A	0,8	142	A	0,6	184	A	0,0	
124	W	0,0	143	W	0,0	185	W	0,0	
125	I	0,0	144	I	0,0	186	I	0,0	
126	E	0,0	145	E	0,0	187	E	0,0	
127	L	0,1	146	L	0,0	188	L	0,1	
128	L	0,1	147	L	0,0	189	L	0,0	
129	I	0,0	148	I	0,0	190	I	0,0	
130	A	0,1	149	A	1,0	191	A	0,0	
131	N	0,0	150	N	0,3	192	N	0,1	
132	V	1,0	151	V	0,0	193	V	0,0	
133	A	1,0	152	A	0,0	194	A	0,0	
134	W	1,0	153	W	0,0	195	W	0,0	
135	C	0,1	154	C	0,0	196	C	0,4	
136	S	0,1	155	S	1,0	197	S	0,0	
137	I	0,1	156	I	0,0	198	I	0,0	
			157	V	0,0	199	V	0,0	
			158	S	0,0	200	S	0,4	
			159	L	0,0	201	L	0,4	
			160	*D*	*0,0*	*202*	*D*	*0,2*	
			161	*D*	*0,9*	*203*	*D*	*0,2*	residues in italics and boxed:
						204	*S*	*0,7*	not stabilised in the crystals
						205	*P*	*0,3*	
						206	*G*	*0,2*	
						207	*L*	*0,0*	
						208	Q	0,0	
			162	P	0,0	209	P	0,0	
			163	Q	0,0	210	Q	0,0	
			164	Q	0,0	211	Q	0,0	
			165	L	0,0	212	L	0,3	ß-s1
			166	F	0,0	213	F	0,3	

138	L	0,1	167	L	0,0	214	L	0,0	
139	N	1,0	168	N	0,9	215	N	0,8	
140	Q	1,0	169	Q	0,0	216	Q	0,2	
141	S	0,0	170	S	0,0	217	S	0,0	
142	F	0,7	171	F	0,0	218	F	0,0	
143	S	0,1	172	S	0,0	219	S	0,4	
144	Y	1,0	173	Y	0,0	220	Y	0,9	ß-s2
145	H	0,0	174	H	0,0	221	H	0,0	
146	R	0,1	175	R	0,0	222	R	0,0	
147	N	0,0	176	N	0,0	223	N	0,0	
148	S	0,1	177	S	1,0	224	S	0,0	
149	A	0,0	178	A	0,0	225	A	0,0	
150	I	1,0	179	I	0,5	226	I	0,5	
151	K	0,0	180	K	0,0	227	K	0,3	
152	A	0,0	181	A	0,0	228	A	0,0	
153	G	0,0	182	G	0,0	229	G	0,1	
154	V	0,0	183	V	0,0	230	V	0,0	
155	S	0,0	184	S	0,0	231	S	0,0	
156	A	0,4	185	A	0,0	232	A	0,0	
157	I	0,0	186	I	0,0	233	I	0,0	
158	F	0,0	187	F	0,0	234	F	0,0	
159	D	0,0	188	D	0,0	235	D	0,0	
160	R	0,1	189	R	0,0	236	R	0,0	LBD H6-H7
161	I	0,1	190	I	0,0	237	I	0,0	
162	L	0,1	191	L	0,0	238	L	0,0	
163	S	0,1	192	S	0,0	239	S	0,0	
164	E	0,0	193	E	0,0	240	E	0,0	
165	L	0,1	194	L	0,0	241	L	0,0	
166	S	1,0	195	S	1,0	242	S	0,0	
167	V	0,9	196	V	0,5	243	V	0,0	
168	K	0,0	197	K	0,0	244	K	0,0	
169	M	0,0	198	M	0,0	245	M	0,0	
170	K	0,0	199	K	0,0	246	K	0,0	
171	R	1,0	200	R	0,1	247	R	0,0	
172	L	0,0	201	L	0,0	248	L	0,0	
173	N	0,6	202	N	1,0	249	N	0,2	
174	L	0,0	203	L	0,2	250	L	0,1	
175	D	0,0	204	D	0,0	251	D	0,0	
176	R	0,4	205	R	0,7	252	R	0,0	
177	R	1,0	206	R	0,0	253	R	0,3	
178	E	0,0	207	E	0,0	254	E	0,0	
179	L	0,1	208	L	0,0	255	L	0,0	
180	S	0,0	209	S	0,4	256	S	0,7	
181	C	0,0	210	C	0,0	257	C	0,9	LBD H8
182	L	0,1	211	L	0,0	258	L	0,0	
183	K	1,0	212	K	0,0	259	K	0,0	

184	A	0,2	213	A	0,0	260	A	0,0	
185	I	0,0	214	I	0,0	261	I	0,0	
186	I	0,0	215	I	0,0	262	I	0,0	
187	L	0,1	216	L	0,0	263	L	0,0	
188	Y	0,0	217	Y	0,0	264	Y	0,0	
189	N	0,0	218	N	0,0	265	N	0,0	
190	P	0,0	219	P	0,0	266	P	0,0	
191	D	0,0	220	D	0,0	267	D	0,0	
192	I	0,1	221	I	0,4	268	I	0,0	
193	R	0,0	222	R	0,0	269	R	0,0	
194	G	0,0	223	G	0,0	270	G	0,0	
195	I	0,1	224	I	0,0	271	I	0,0	
196	K	0,0	225	K	0,0	272	K	0,0	
197	S	1,0	226	S	0,5	273	S	0,0	
198	R	1,0	227	R	0,0	274	R	0,0	
199	A	0,0	228	A	0,0	275	A	1,0	
200	E	0,0	229	E	0,0	276	E	0,1	
201	I	0,1	230	I	0,0	277	I	0,0	
202	E	0,8	231	E	0,0	278	E	0,2	
203	M	1,0	232	M	0,0	279	M	0,0	
204	C	0,1	233	C	0,0	280	C	1,0	
205	R	0,1	234	R	0,0	281	R	0,0	
206	E	0,0	235	E	0,0	282	E	0,0	
207	K	0,0	236	K	0,0	283	K	0,0	
208	V	0,1	237	V	0,0	284	V	0,0	LBD H9
209	Y	0,0	238	Y	0,0	285	Y	0,0	
210	A	0,0	239	A	0,0	286	A	0,0	
211	C	1,0	240	C	0,0	287	C	0,0	
212	L	0,1	241	L	0,0	288	L	0,0	
213	D	0,3	242	D	0,0	289	D	0,0	
214	E	0,0	243	E	0,0	290	E	0,0	
215	H	0,3	244	H	0,0	291	H	0,0	
216	C	1,0	245	C	0,0	292	C	0,0	
217	R	0,0	246	R	0,0	293	R	0,1	
218	L	0,0	247	L	0,0	294	L	0,0	
219	E	0,9	248	E	0,3	295	E	0,1	
220	H	0,0	249	H	0,5	296	H	0,0	
221	P	0,0	250	P	0,7	297	P	0,0	
222	G	0,9	251	G	0,0	298	G	0,8	
223	D	0,0	252	D	0,0	299	D	0,2	
224	D	1,0	253	D	0,0	300	D	0,0	
225	G	0,0	254	G	0,0	301	G	0,0	
226	R	0,1	255	R	0,0	302	R	0,0	
227	F	0,0	256	F	0,0	303	F	0,0	
228	A	0,0	257	A	0,0	304	A	0,0	
229	Q	1,0	258	Q	0,4	305	Q	0,0	

230 L	0,1	259 L	0,0	306 L	0,0	
231 L	0,1	260 L	0,0	307 L	0,0	
232 L	0,1	261 L	0,0	308 L	0,0	
233 R	0,0	262 R	0,0	309 R	0,0	
234 L	0,1	263 L	0,0	310 L	0,0	
235 P	0,0	264 P	0,0	311 P	0,0	
236 A	0,0	265 A	0,0	312 A	0,0	
237 L	0,1	266 L	0,0	313 L	0,0	
238 R	0,0	267 R	0,0	314 R	0,0	LBD H10-H11
239 S	0,0	268 S	0,0	315 S	0,0	
240 I	0,0	269 I	0,0	316 I	0,0	
241 S	1,0	270 S	0,0	317 S	0,0	
242 L	0,1	271 L	0,0	318 L	0,0	
243 K	0,0	272 K	0,0	319 K	0,0	
244 C	0,0	273 C	0,0	320 C	0,0	
245 Q	0,1	274 Q	0,3	321 Q	0,0	
246 D	0,0	275 D	0,3	322 D	0,0	
247 H	0,0	276 H	0,0	323 H	0,0	
248 L	0,1	277 L	0,0	324 L	0,0	
249 F	0,0	278 F	0,0	325 F	0,0	
250 L	0,0	279 L	0,0	326 L	0,0	
251 F	0,0	280 F	0,0	327 F	0,0	
252 R	0,0	281 R	0,0	328 R	0,0	
253 I	0,1	282 I	0,0	329 I	0,0	
254 T	0,0	283 T	0,2	330 T	0,0	
255 S	0,0	284 S	0,0	331 S	0,0	
256 D	0,0	285 D	0,0	332 D	0,0	
				333 R	0,3	
258 P	0,0	286 P	0,0	334 P	0,7	
259 L	0,1	287 L	0,6	335 L	0,0	
260 E	0,3	288 E	0,0	336 E	0,0	
261 E	0,1	289 E	0,6	337 E	0,9	LBD H12
262 L	0,0	290 L	0,4	338 L	0,3	
				339 F	0,0	
				340 L	0,1	
				341 E	0,0	
				342 Q	0,4	
				343 L	0,0	
				344 E	0,2	
				345 A	0,1	
				346 P	0,0	

Table S6
