# Supplementary material for: Molecular adaptation and resilience of the insect’s nuclear receptor USP
Source: BMC Evol Biol. 2012 Oct 5;12:199. doi: 10.1186/1471-2148-12-199 (PMC3520820; doi:10.1186/1471-2148-12-199)
Supplement: Additional file 9 — Table S2. Relative synonymous codon usage (RSCU). [file 1471-2148-12-199-S9.doc]

| **Amino acid** | **Codon** | **Diptera** | **Lepidoptera** | ***Tribolium*** | **Blattaria** |
| --- | --- | --- | --- | --- | --- |
| **Leu** | CT**C** | **0,84** | **1,60** | **1,13** | 0,78 |
|  | CT**G** | **2,87** | **2,72** | 0,51 | 1,23 |
|  | TT**G** | **0,84** | 0,57 | **2,33** | **1,47** |
|  | CTT | 0,36 | 0,31 | 0,69 | **2,05** |
|  | CTA | 0,38 | 0,53 | 0,40 | 0,31 |
|  | TTA | 0,27 | 0,28 | 0,95 | 0,17 |
| **Phe** | TT**C** | **1,33** | **1,80** | **1,03** | 0,86 |
|  | TTT | 0,67 | 0,20 | 0,97 | **1,14** |
| **Ala** | GC**G** | **0,98** | **2,24** | 0,98 | 0,52 |
|  | GC**C** | **1,82** | **1,11** | **1,16** | 0,72 |
|  | GCA | 0,46 | 0,18 | 0,84 | **1,07** |
|  | GCT | 0,73 | 0,46 | **1,02** | **1,66** |
| **Tyr** | TA**C** | **1,26** | **1,66** | **1,37** | **1,36** |
|  | TAT | 0,74 | 0,34 | 0,63 | 0,64 |
| **Val** | GT**G** | **1,69** | **2,05** | **1,17** | **1,09** |
|  | GT**C** | **1,08** | **0,87** | **1,40** | 0,41 |
|  | GTA | 0,50 | 0,64 | 0,68 | 0,57 |
|  | GTT | 0,73 | 0,44 | 0,75 | **1,93** |
| **Glu** | GA**G** | **1,49** | **1,33** | 0,83 | **1,07** |
|  | GAA | 0,51 | 0,67 | **1,17** | 0,93 |
| **Gln** | CA**G** | **1,45** | **1,38** | 0,70 | **1,60** |
|  | CAA | 0,55 | 0,63 | **1,30** | 0,40 |
| **Ser** | TC**C** | 1,09 | 1,09 | **1,66** | 0,64 |
|  | TC**G** | **1,44** | **1,78** | **2,90** | 0,30 |
|  | AG**C** | **1,61** | **1,33** | 0,00 | 0,84 |
|  | TCT | 0,25 | 0,66 | 0,41 | **1,54** |
|  | TCA | 0,54 | 0,57 | 1,03 | **1,64** |
|  | AGT | 1,07 | 0,57 | 0,00 | 1,04 |
| **Arg** | CG**G** | **1,47** | **1,17** | 1,04 | 0,29 |
|  | CG**C** | **2,33** | **2,10** | 0,21 | **1,93** |
|  | AG**G** | 0,33 | 0,84 | **1,88** | 0,64 |
|  | CGA | 0,83 | 0,90 | 0,99 | **1,54** |
|  | CGT | 0,79 | 0,50 | 0,47 | 1,18 |
|  | AGA | 0,24 | 0,50 | **1,41** | 0,43 |
| **Thr** | AC**G** | 0,98 | **1,80** | **1,37** | 0,71 |
|  | AC**C** | **1,40** | **1,27** | 0,44 | 0,68 |
|  | ACA | **1,23** | 0,60 | **1,41** | **1,82** |
|  | ACT | 0,38 | 0,33 | 0,78 | **0,79** |
| **Cys** | TG**C** | **1,32** | **1,25** | 0,71 | **1,12** |
|  | TGT | 0,68 | 0,75 | **1,29** | 0,88 |
| **Lys** | AA**G** | **1,29** | **1,29** | 0,71 | **1,24** |
|  | AAA | 0,71 | 0,71 | **1,29** | 0,76 |
| **Asn** | AA**C** | **1,16** | **1,40** | **1,17** | 0,96 |
|  | AAT | 0,84 | 0,60 | 0,83 | **1,04** |
| **Pro** | CC**G** | **1,92** | **1,18** | **1,07** | 0,95 |
|  | CC**C** | **0,88** | **1,15** | 0,71 | 0,51 |
|  | CCA | 0,76 | 0,85 | 0,89 | **1,31** |
|  | CCT | 0,44 | 0,82 | **1,33** | **1,24** |
| **Asp** | GA**C** | 0,91 | **1,58** | 0,81 | 0,71 |
|  | GAT | **1,09** | 0,42 | **1,19** | **1,29** |
| **Gly** | GG**G** | 0,54 | 0,69 | 0,80 | 0,68 |
|  | GG**C** | **1,89** | **1,82** | **1,09** | **1,79** |
|  | GGA | 0,66 | 0,69 | **1,16** | 0,57 |
|  | GGT | **0,91** | **0,79** | 0,95 | **0,96** |
| **His** | CA**C** | 0,81 | **1,46** | 0,74 | 0,44 |
|  | CAT | **1,19** | 0,54 | **1,26** | **1,56** |
| **Ile** | AT**C** | **1,68** | **1,69** | **1,29** | **1,16** |
|  | ATT | 0,73 | 0,32 | 0,61 | 0,69 |
|  | ATA | 0,58 | 0,99 | 1,10 | **1,16** |

**Table S2.** Relative synonymous codon usage (RSCU). Preferred codons are in bold and underlined.
