# Supplementary material for: Molecular adaptation and resilience of the insect’s nuclear receptor USP
Source: BMC Evol Biol. 2012 Oct 5;12:199. doi: 10.1186/1471-2148-12-199 (PMC3520820; doi:10.1186/1471-2148-12-199)
Supplement: Additional file 10 — Table S7. Degenerated primers used to clone usp and cox1 genes. [file 1471-2148-12-199-S10.doc]

| **Drosophilidae USP Primers:** |
| --- |
| CAGTATCCNCCNAAYCAYCC U50dbis |
| GGNAARCAYTAYGGNGTNTACAG U51dbis |
| GGCATGAARCGNGARGCGGTNCA U53dbis |
| CTCCGACAANATNCGRTCRAA U33dbis |
| CTGATNGANCGCAANGCNGG U31dbis |
| GATACGGCGCCCTTGTAGTC USPREV1 |
| TTCTTAATCAGAACTTCTCATATCATC CONFUSPAV1 |
| TCATATCATCGCAACAGCGCCATC CONFUSPAV2 |
| GTCAAGCCGGAYATHTCNCA USPDIR |
| CGNWAGGCCGGGNGGNGG USPFIN |
| GANACGGCNCCCTTGTARTC USPREVU |
| ATCAARGARGARGTNAARCC USPDIR0 |
| GCTGTCCTGCCTGAAGGC USPDIR2 |
| AGGCSATCATACTGTACAA USPDIR3 |
| GGCGATCGTGCACTGACGTT SGMELUSPDIR |
| GACAGCAACAACAGCAGCTTTTC SGMELUSPDEB |
| TCGGGCGTTGGCATTTCTGC HYDUSP1 |
| TCTCGCGGCACAGCTCGATG HYDUSP2 |
| GGCACAGCTCGATATCGGC VIRUSPREV |
| CCGTGACTTTACCATCGA WILLUSP1 |
| CGTCTAGACAGGCGTAGACC WILLUSP2 |
| GCTGTCCTGCYTGAARGC USPDIR2U |
| ATCGAACGGCTGATGGACGC USPANADIR |
| CACAGTGCGCAAGGATCTCACATA U52dbis |
| TCGGCGCGATTCTTGATGC VIRUSPREV2 |
| TGYTCNAGGCGRCARTGYTCRTC USPSECHREVU |
| CAGTATCCGCCTAACCATCC USPSECHDIR |
| GGCKGCRMYTCNAGYTGYTC USPFINBIS |
| CGGGCCAGTGGCAAGCACT VIRUSPDIR |
| **Drosophilidae COX1 primers:** |
| CARTGRTTRTTYTCNACNAAYCA COIDIR |
| ATCTACAGAAGCTCCTCCAT COIREVHYDEI |
| CCTTTTTACCTGATTCTTGAC COIREVWILLI |
| GCTCATAAAATAGCTGGAGA COIREVPSEU |
| AATTCAGAATATCTATGYTC COIREVFIN |
| TACCAACATTTATTTTGATT COIDIRHYDEI |
| GGAGTAGTTTTAGCTAATTC COIDIRWILLI |
| GTAAACCTAAATTAGCTCA CO2REVDEB |
| CACCCAGGAGCTTTAATTGG COICARIDIR |
| ***Tribolium* USP primers:** |
| - forward (usp51): GGIAA(a/g)CA(c/t)TA(c/t)GGIGTITACAG |
| - forward (usp53): GGIATGAAG(a/c)GIGA(a/g)GC |
| - reverse (usp38): IATIAGCA(a/g)(c/t)TC(a/g)TTCCA |
| - reverse (usp31): GTGTCICCIATIAG(c/t)TT(a/g)AA |
| - reverse (usp30) : GA(c/t)TCIA(a/g)CATCTCCA(c/t)SAG(a/g)AA |
| - reverse (ecr31): (c/g)IA(c/t)(a/g)TCCCA(a/g)A(c/t)(c/t)TCITCIA(a/g)GAA |
| ***Tribolium* COX1 primers:** |
| - forward (co50) : TC(A/T)AC(A/T)AA(C/T)CA(C/T)AAAGATATT |
| - forward (co51) : TA(C/T)CAACAC(C/T)TATTCTGATT |
| - reverse (co30) : TCAGA(A/G)TA(A/T)CT(A/G)TGTTC(A/T)GC) |
| - reverse (co31) : (A/G)GTTTACTCCIATGAATAT |
| - reverse (co32) : TTAG(A/G)AATTG(G/T)(A/G)(C/T)(C/T)TTACATA |
| **Blattaria USP primers:** |
| forward (USPBOXF): 5’- TAYCCNCCNAAYCAYCC-3’ |
| reverse (PUSPR1): 5’-GGCAGACGTAGAAGCAGTTTG-3’ |
| **Blattaria COX1 primers** |
| forward (COIF2): 5’- ACWAATCATAAAGATATTGGNACNYT-3’ |
| reverse (COIR2): 5’-GGATAATCWSWATAWCGWGGYAT-3’ |

**Table S7.** Degenerated primers used to clone *usp* and *cox1* genes.
